# Assessing verbal and spatial memory via smartphone

**DOI:** 10.3389/fdgth.2026.1680389

**Published:** 2026-03-10

**Authors:** Peter Kosa, Amir Moghadam Ahmadi, Marie Kanu, Yolanda Mejia, Darwing Padilla-Rolon, Bibiana Bielekova

**Affiliations:** Neuroimmunological Diseases Section, Laboratory of Clinical Immunology and Microbiology, National Institute of Allergy and Infectious Diseases, National Institutes of Health, Bethesda, MD, United States

**Keywords:** cognitive decline, digital biomarkers, memory testing, multiple sclerosis, phone app

## Abstract

**Introduction:**

Detecting subtle cognitive decline in chronic central nervous system (CNS) disease is hampered by practice effects, motor confounds, and the lack of premorbid baselines. Smartphone testing offers frequent unsupervised assessments but requires rigorous validation and internal quality control. Therefore, we aimed to develop and validate smartphone-based verbal and spatial memory tasks, derive composite digital biomarkers, and determine their utility for monitoring cognitive impairment.

**Methods:**

In a prospective study (2018–2025) we enrolled 315 adults [41 healthy donors, 217 people with Multiple Sclerosis (MS), 57 other CNS disorders]. Participants completed 5,875 verbal and 2,588 spatial trials on the Neurological Functions Test Suite App. We extracted fourteen digital biomarkers reflecting individualized Sensory-Motor Processing Thresholds (iSMPT), adjusted test latencies and test accuracies, and split the MS cohort into training (*n* = 145) and validation (*n* = 72) sets. Ordinal logistic regression generated composite scores that were tested in the held-out cohort. Reliability (intraclass correlation coefficient, ICC) and criterion validity (Spearman Rho, R², Lin's concordance) were calculated; *p*-values < 0.05 were considered significant.

**Results:**

The spatial memory composite separated healthy donors from relapsing-remitting multiple sclerosis (RR-MS), progressive MS, non-inflammatory and inflammatory neurological diseases, and radiologically/clinically isolated syndromes (all *P* < 0.05), outperforming all single biomarkers. With iSMPT included, it correlated most strongly with global disability [Expanded Disability Status scale [EDSS], Neurological exam composite [NeurEx]; Rho = 0.62–0.63, *P* < 0.001] and lesion volume (Rho = 0.71, *P* = 0.008); without iSMPT it tracked cognitive subscores [EDSS Functional System Score 7 (FSS7), NeurEx panel 1; Rho = 0.50–0.53, *P* < 0.001] and Combinatorial MRI Score of CNS tissue destruction (COMRIS-CTD; Rho = 0.63, *P* < 0.001). Test–retest reliability was good (ICC = 0.813 with iSMPT; 0.713 without). A linear model using spatial memory biomarkers predicted Symbol Digit Modalities Test (SDMT) scores in an independent cohort (*n* = 63; R² = 0.53, Rho = 0.74, concordance = 0.66), enabling a 95% prediction envelope for real-time quality control.

**Discussion:**

The spatial memory composite provides a rapid, reliable, and sensory-motor delay-adjusted digital marker of mild cognitive dysfunction. Coupled with SDMT, it enhances unsupervised monitoring and internal quality assurance across diverse CNS disorders.

## 1 Introduction

Cognitive impairment is one of the most common and often most disabling consequences of central nervous system (CNS) diseases. Severe deficits are easy to spot during a standard neurological examination, yet subtle decline is notoriously hard to detect and even harder to track over time. Accurate longitudinal measurement matters: it underpins decisions about disease progression, therapeutic response, and rehabilitation. The difficulty arises because mild decline must be judged against an individual's premorbid ability—information that is seldom available and usually inferred from imperfect proxies such as education or occupation. Additionally, performance on traditional neurocognitive tests is affected by practice effects and non-cognitive influences, such as motivation, fatigue, mood, medications and sleep quality (1). Therefore, identifying mild cognitive impairment requires repeated administration of standardized assessment batteries, often tailored to specific disease (2).

Multiple sclerosis (MS), chronic inflammatory-demyelinating CNS disease illustrates this challenge. Unlike the amnestic-cortical profile of cognitive impairment seen in primary dementias such as Alzheimer's disease, the most prevalent deficit in MS is slowed information processing speed (IPS) (2, 3), caused by multiple deep white matter (WM) and juxtacortical/cortical lesions resulting in disconnection (axonal transections) or delay (demyelination) of synaptic circuits. Other commonly affected domains include working and episodic memory, both verbal and visuospatial (4).

MS-tailored standardized assessment cognitive batteries evolved over decades of consensus-based research from the Brief Repeatable Battery of Neuropsychological Tests (BRB-N) that took approximately 45 min to administer, to a more comprehensive, 90-minute MACFIMS battery [i.e., The Minimal Assessment of Cognitive Functions in MS (5, 6)] to finally, a more practical and internationally adopted BICAMS (i.e., the Brief International Cognitive Assessment for MS) battery (2, 7). BICAMS takes ∼15 min to administer and includes three most successful cognitive tests from MACFIMS: 1. the Symbol Digit Modalities Test (SDMT), which gauges IPS, attention and concentration; 2. the California Verbal Learning Test-II (CVLT-II) which assesses verbal memory; and 3. the Brief Visuospatial Memory Test-Revised (BVMT-R), which measures visuospatial memory. While all three tests have few equivalent forms allowing 2–3 repeated administrations, none is sufficiently randomized to prevent memorization in long-term cognitive monitoring spanning chronic diseases like MS. Of these tests, the SDMT emerged as sentinel for MS-associated cognitive impairment (8), and the most successful test in MS clinical trials (9–11).

In its conventional paper SDMT format, participants consult a fixed key that pairs nine symbols with nine digits, then match as many symbols to digits as possible in 90 s. We recently created a smartphone SDMT that randomizes both the key and the symbol sequence at every sitting, preventing memorization and allowing truly longitudinal assessment (12). The mobile test takes less than five minutes, and it matches or exceeds the psychometric properties of the paper version, and correlates strongly with volumetric magnetic resonance imaging (MRI) measures of tissue damage. After adjusting for motor and visual impairment, SDMT performance alone explained 75% of the variance in MRI-visible brain injury in an independent cohort. Longitudinally, its intraclass correlation coefficient (ICC) reached 0.90 once an initial learning phase was excluded. Even so, a decline of 13 SDMT points (roughly a 15%–30% performance drop) was required to declare genuine cognitive worsening on a patient level. Averaging four weekly baseline trials reduced that threshold to seven points, underscoring the inherent variability of even the best single cognitive test and highlighting the value of frequent, repeated assessments.

The smartphone SDMT forms part of the Neurological Functions Test Suite (NeuFun-TS), an initiative to recreate key elements of the neurological examination on a patient's own device. NeuFun-TS is intended both as a screening tool, flagging individuals who might benefit from specialist referral, and as a monitoring platform for clinical trials and routine care. Each NeuFun-TS module generates digital biomarkers that are benchmarked against clinician-derived disability scales and neuro-imaging outcomes. Promising metrics are combined into composite scores to enhance reliability, whereas underperforming tests are refined or retired (12–16).

Because NeuFun-TS is self-administered and unsupervised, robust internal quality control is essential. Our strategy is to interrogate each neurological domain with at least two independent tasks and to use correlation-based prediction intervals to identify unreliable results. Guided by this principle, the aim of this study was to design alternative cognitive tests within NeuFun-TS that could both broaden and strengthen the identification of cognitive deficit in people with MS (pwMS), while simultaneously providing a means to internally cross-validate novel tests with the SDMT.

In view of the frequently affected cognitive domains in pwMS and building on the work that led to BICAMS, we initially considered adapting the BICAMS memory tests for smartphone use. However, we found that their execution (e.g., the hand-drawing required for the BVMT-R) and subsequent scoring were unsuitable for frequent, unsupervised administration and algorithmic scoring via smartphones. We therefore adopted a Paired-Associates Learning paradigm (17) for verbal memory. For non-verbal/visuospatial memory we adopted the Visual Matrix Task/Visual Patterns Test (VMT/VPT (18, 19)). Additionally, since visuospatial memory comprises two distinct circuits (18, 20), visual memory (static visuospatial memory, measured by VMT/VPT) and spatial memory [dynamic visuospatial memory (19), conventionally measured by the Corsi block-tapping task (21)], we developed a novel dynamic adaptation of VMT/VPT, as the Corsi block-tapping task is not easily amenable to smartphone adaptation. This paper describes the derivation of digital biomarkers from these tests, examines their psychometric performance, and shows how they extend, and help validate the flagship smartphone SDMT.

## 2 Materials and methods

### 2.1 Study design and subjects

We conducted a prospective, observational study under two National Institutes of Health (NIH) protocols: NCT00794352 (“Comprehensive Multimodal Analysis of Neuroimmunological Diseases of the CNS”) and NCT03109288 (“TRAP-MS: Targeting Residual Activity by Precision, Biomarker-Guided Combination Therapies of MS”) to evaluate novel smartphone-based tests of verbal and spatial memory. The Institutional Review Board of the NIH approved both protocols, and all participants provided written or electronic informed consent before any study procedures began. Data collection spanned from January 26, 2018 (the date we rolled out our first smartphone test) through February 4, 2025; every subject with at least one valid NeuFun-TS assessment in that interval was included. Because the verbal memory module launched on January 26, 2018, over three years before the spatial memory test (July 28, 2021), the verbal cohort is correspondingly larger. Demographics and total data point counts are provided in Table 1 and Supplementary Figures S1, S2.

**Table 1:** Demographic data.

Verbal memory test								
Diagnosis	HD	NIND	OIND	RIS	CIS	RR-MS	SP-MS	PP-MS
*Subjects* (*n*)	34	29	26	2	7	96	61	60
*Female sex* (*n*, %)	17 (50%)	21 (72.4%)	17 (65.4%)	1 (50%)	6 (85.7%)	64 (66.7%)	40 (65.6%)	35 (58.3%)
*Trials per subject* median (Q1–Q3), min-max	12 (2–20), 1–22	2 (2–4), 1–13	3 (2–5), 1–9	3 (2–4), 2–4	4 (2–9), 2–10	5 (3–8), 1–390	7 (2–12), 1–1,238	6 (2–12), 1–382
*Age at first trial* median (Q1–Q3), min-max	46.4 (25.2–55.6), 19.2–79.4	46.9 (38–57.8), 24.9–79.1	43 (34.6–54.8), 21.4–71.9	54.6 (52.4–56.9), 50.1–59.1	40.5 (31.8–44.3), 25.7–64.2	46.9 (36.5–54.7), 21.9–78.6	59.2 (49.6–65.2), 28.1–75	61 (53.7–65.9), 19.4–74.9
*EDSS at first trial*	2 (1.5–3), 1–3.5	3.5 (2–4.5), 1–8	4.2 (3.5–6), 1–8	3.2 (2.6–3.9), 2–4.5	2.5 (1.8–3), 1.5–6.5	3.5 (2.5–4.5), 1.5–6.5	6.2 (5.5–6.5), 2.5–8	6 (5–6.5), 3–8
*NeurEx at first trial* median (Q1–Q3), min-max	28.2 (13.5–36.2), 1–67.7	61.8 (27.5–82.8), 9.6–218.2	80.6 (51.5–137.3), 2–417.4	49.4 (36.4–62.3), 23.5–75.2	43.3 (16.2–51.6), 4.5–186.6	60.7 (40.8–90.2), 7.1–251.4	192.2 (143.1–232.2), 45.3–517.7	167.8 (111–215), 40.7–520.8
*SDMT at first trial* median (Q1-Q3), min-max	64.8 (59.8–75), 34–94.2	55.8 (43.8–60), 4–77.4	43.8 (35.2–51.3), 21–62	39.5 (39.2–39.8), 39–40	55.8 (51.2–61.8), 46.2–72.6	51 (44–58.2), 5–83	37.9 (30.1–48.6), 13–66.6	42.6 (34–50.5), 13.8–75
Spatial memory test								
Diagnosis	HD	NIND	OIND	RIS	CIS	RR-MS	SP-MS	PP-MS
*Subjects* (*n*)	31	22	16	1	6	78	44	42
*Female sex* (*n*, %)	16 (51.6%)	16 (72.7%)	12 (75%)	1 (100%)	5 (83.3%)	54 (69.2%)	29 (65.9%)	25 (59.5%)
*Trials per subject* median (Q1–Q3), min-max	12 (2–20), 2–25	2 (2–4), 2–8	2 (2–4), 2–8	4 (4–4), 4–4	6 (2–8), 2–8	4 (2–8), 2–196	6 (2–10), 1–545	4 (2–8), 2–50
*Age at first trial* median (Q1–Q3), min-max	43.9 (24.9–53.7), 19.2–79.4	48 (38.6–59.8), 26.1–79.1	40.5 (30.3–44.3), 21.4–66.5	50.1 (50.1–50.1), 50.1–50.1	41.4 (38.3–46.9), 26.3–64.2	47.4 (37.2–55.8), 23–80.5	61 (52–67.9), 32.9–74.9	61.8 (56.1–67.1), 21.7–77.8
*EDSS at first trial* median (Q1–Q3), min-max	2 (1.5–3), 1–3	3.5 (2.1–4.4), 1–5	4 (2.9–6), 1–6.5	4.5 (4.5–4.5), 4.5–4.5	2.2 (1.6–2.9), 1.5–6.5	3.5 (3–4.5), 1.5–6.5	6 (5.5–6.5), 3–8	5.8 (5–6.5), 3–8
*NeurEx at first trial* median (Q1–Q3), min-max	22.8 (13.5–33.3), 5.2–49.2	62 (35.1–81.3), 9.6–141.5	79.3 (44.6–110.6), 2–156.4	75.2 (75.2–75.2), 75.2–75.2	32.4 (19.8–43.8), 12.9–186.6	64.1 (48.8–95), 16.1–236.4	188.2 (136.2–240.2), 47.6–375.4	153 (113.2–216.5), 40.7–558.9
*SDMT at first trial* median (Q1–Q3), min-max	66.6 (63–85.8), 39–94.2	58.2 (47.4–60.6), 23.4–77.4	48.6 (43.5–53.7), 21–59.4	39 (39–39), 39–39	54.6 (48–63.9), 46.2–79.8	53.4 (46.2–61.8), 24.6–89.4	40.2 (30.6–51.9), 11.4–73.8	41.7 (38.1–54), 13.8–75

*n* = sample size, Q1/3 = first/third quartile, min, minimum; max, maximum; HD, healthy donor; NIND, non-inflammatory neurological disease; OIND, other inflammatory neurological disease; RIS, radiologically isolated syndrome; CIS, clinically isolated syndrome; RR-MS, relapsing-remitting multiple sclerosis; SP-MS, secondary progressive MS, PP-MS, primary progressive MS.

Participants fell into three NeuFun-TS cohorts based on how and where they completed the tests. Cohort 1 consisted of healthy donors (HD) who didn't want to participate in all aspects of the Natural history protocol (e.g., lumbar puncture, MRI, formal neurological exam); they consented to “smartphone only cohort” via NeuFun-TS App and performed all NeuFun-TS tasks unsupervised on investigator-provided smartphones. Cohort 2 included subjects who completed NeuFun-TS tests in person at the NIH Clinical Center, typically within one day of a full neurological evaluation and brain MRI. Finally, Cohort 3 comprised a subset of Cohort 2 participants who opted to continue home testing; they received weekly “prescriptions” for NeuFun-TS assessments and performed the tests at their convenience.

### 2.2 Clinical outcomes

Neurological exams were recorded in real time via NeurEx™ App (22) on iPads or laptop/desktop computers. NeurEx™ App serves as a digital Case Report Form as it defines all aspects of neurological examination that must be scored: the NeurEx™ App consists of 17 pages, each corresponding to a domain of the neurological examination where a clinician documents identified deficits using touch and swipe gestures on homunculus-like representations. The App algorithmically translates documented neurological examination into traditional disability scales used in neuroimmunology, including the Expanded Disability Status Scale (23) (EDSS, ordinal scale from 0 to 10), and associated Kurtzke Functional System Scores [FSS], which include FSS of cerebral/mental functions (FSS7). Additionally, the NeurEx™ App scores all imputted data as a continuous disability scale (NeurEx) from 0 (no disability) to theoretical maximum of 1,349. NeurEx subscores can be generated for any disability domain (e.g., cognitive functions; NeurEx1 subscore). Smartphone SDMT scores were collected through the validated NeuFun-TS test (15).

### 2.3 Imaging outcomes

Clinic-associated brain MRIs extending to the upper cervical spinal cord C5 level were used to generate Combinatorial MRI scale of CNS tissue destruction (COMRIS-CTD) score derived from a machine-learning model as previously described (24). COMRIS models predict current scales of neurological disability used in MS research with strong performance in the independent validation cohort (25).

The volumetric MRI data of different CNS structures were computed by the LesionTOADS algorithm (26) implemented in a cloud based platform QMENTA (https://www.qmenta.com), generating volume data for 12 different segmented CNS tissues (e.g., lesion volume, ventricular volume). Brain parenchymal fraction (BPFr) was calculated as a ratio between brain parenchyma volume and the total intracranial volume.

### 2.4 App development, data collection and processing

**Figure 1:**
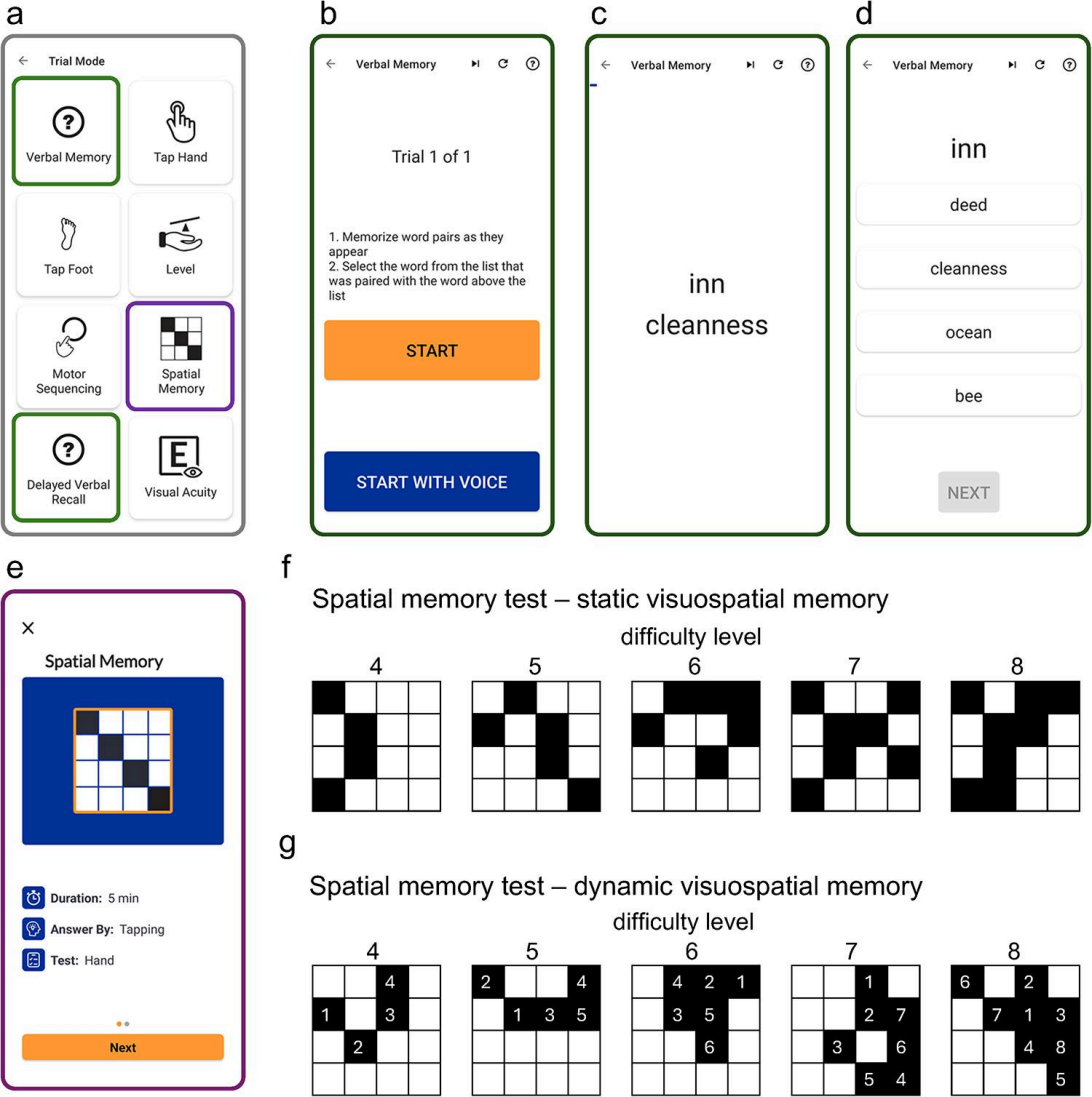
Design of the verbal and spatial memory tests in the NeuFun-TS App. **(a)** Dashboard showing the immediate and delayed verbal memory tests (green) and the spatial memory test (purple). **(b)** Verbal memory instructions. **(c)** Presentation phase: ten word pairs appear sequentially for 5 s each. **(d)** Recall phase: each cue word is shown with four choices and participants select its pair. **(e)** Spatial memory initial screen. **(f)** Static stage: participants view a 4 × 4 grid pattern of black squares for 5 s, then recreate it in an empty grid; correct reproductions advance the difficulty level, incorrect attempts are repeated, until five trials are completed. **(g)** Dynamic stage: participants memorize both the spatial pattern and the serial order of square presentations before reconstruction; this stage also comprises five trials.

We built the NeuFun-TS memory tests in Kotlin/Java using Android Studio, optimizing the interface for Google Pixel XL and 2 XL devices. Both verbal and spatial tests reside within the NeuFun-TS App's home screen (Figure 1a) and upload results in real time to a secure Firebase database.

The details of memory tests (Figure 1) will be described in the Results. The introductory screens for each test explain the test in detail and each subject has an opportunity to do “test trial” to assure complete comprehension of the tasks.

All raw NeuFun-TS outputs streamed in real time to private database hosted on Google Firebase. We deployed custom extraction scripts to pull JSON logs for each trial, capturing timestamps, correct/incorrect flags for word pair selections (verbal memory), and grid-cell toggles (spatial memory). All raw JSON logs are linked to pseudonymized subject IDs within Firebase and transferred securely to National Institute of Allergy and Infectious Diseases (NIAID) encrypted servers for scoring, quality control, and integration with clinical and imaging data. After integrity checks (e.g., completion time, missing data), we computed trial scores according to algorithms detailed in the Results.

### 2.5 Mathematical modeling and statistical methods

The datasets were split into a training (2/3) and validation cohort (1/3) using *rsample* package (https://rsample.tidymodels.org/), with stratification based on SDMT outcomes to balance levels of cognitive disability across cohorts. The training cohort consisted of up to 10 consecutive trials per participant, whereas the validation cohort used only the first available trial per participant.

To obtain a conservative and unbiased estimate of real-world performance, we intentionally restricted the validation cohort to a single (first) trial per participant. This design mirrors the intended clinical deployment of NeuFun-TS, where early results, often obtained before extensive practice, are most relevant for interpretation and decision-making. In contrast, including multiple trials per participant in the validation cohort would risk inflating performance estimates due to practice effects and within-subject averaging. As demonstrated in the training analyses, model performance improves when multiple trials per participant are averaged, indicating that the reported validation metrics likely represent a lower-bound estimate of performance rather than an overestimate. This approach prioritizes generalizability and robustness over optimistic accuracy estimates.

Ordinal logistic regression models were generated in the training cohort using *caret* package (27) with 10-fold cross-validation to select the best method based on cross-validation model accuracy. The final model was fitted using *MASS::polr* function (28) and evaluated in the independent validation cohort. Multiple linear regression models predicting SDMT were generated using *stats::lm*, followed by stepwise model selection with *MASS::stepAIC* (28), and subsequently tested in the validation cohort.

Datasets lacking normal distribution were log_10_-transformed. Spearman's rank-order correlation coefficient (Rho) was used to evaluate associations between variables. Linear fit's coefficient of determination (R²), its *p*-value (*P*) and sample size (*n*) were also reported. Lin's concordance correlation coefficient (CCC) was reported for comparisons between measured and predicted outcomes. Differences between groups of samples or a null-hypothesis of 0 change within a group of patients were tested using Wilcoxon rank-sum test (two-groups), or Wilcoxon signed-rank test (paired samples) and raw *p*-values are shown. Reliability of longitudinal measurements among patients from different diagnostic groups was assessed by calculating ICC from random-intercept linear mixed-effects models using the *lme4* package (29).

Incomplete or invalid trials were excluded at the trial level based on predefined integrity criteria (e.g., incomplete execution or implausible timing), without subject-level imputation. All analyses were performed in R Studio running R version 4.3.3 (30).

Ninety-five percent confidence intervals (CIs) are reported for all key effect size metrics (Supplementary Tables S1–S11). ICC estimates are reported with 95% CIs derived from the underlying random-intercept mixed-effects models. For correlation and regression analyses, 95% CIs for Spearman's rank correlation coefficient (Rho) and linear-model coefficient of determination (R²) were estimated using nonparametric bootstrap resampling at the participant level (2,000 iterations). For ordinal logistic regression models, odds ratios are reported with Wald-type 95% confidence intervals and corresponding Wald *p*-values. Complete-case analysis was used for all CI computations.

The raw dataset for verbal and spatial memory is available as Supplementary File S1. The R code is available as Supplementary Data Sheet 1.

## 3 Results

### 3.1 Development and implementation of NeuFun-TS memory modules

During the verbal memory learning phase, participants view ten word pairs on the smartphone screen (each displayed for five seconds) and form associative links (Figures 1b,c). Immediately afterwards, they enter the immediate recall stage: for each cue word, they choose its partner from four options displayed on the screen (Figure 1d). Perfect performance (10/10) ends the trial; any error triggers repeated trial. Following a 20-minute break during which other NeuFun-TS tasks are completed, the delayed recall stage repeats the same procedure. The app logs each tap's timestamp, the cue and selected words, and whether the response was correct.

Our spatial module is a novel two-stage task designed to probe pattern recognition (static visuospatial memory) and sequential encoding (dynamic visuospatial memory) on a 4 × 4 grid (Figure 1e, Supplementary Figure S3a). In the static stage, participants study randomly generated checker-board patterns that increase in difficulty (4–8 squares; adjacent by at least a corner, Figure 1f) for five seconds. The pattern then vanishes, and users reconstruct it by tapping grid squares. Correct responses advance the user to the next level (e.g., from 4 square pattern to 5 square pattern), while errors prompt a new pattern of the same complexity. Scores sum the total squares correctly placed (maximum 30 points: 4 + 5 + 6 + 7 + 8).

The dynamic stage adds a temporal component: participants not only replicate each static pattern but must reproduce the exact sequence of square placements (Figure 1g, Supplementary Figure S3b). Five patterns of increasing complexity (4–8 squares) each carry double weight, yielding up to 60 points (8 + 10 + 12 + 14 + 18) for flawless performance. Failed attempt triggers repeating the same difficulty level with a new pattern, keeping the total number of attempts to five. As with the verbal test, every tap (i.e., selection or deselection) is timestamped.

### 3.2 Cohort description and training/validation split

Across all cohorts, we collected a total of 3,364 immediate verbal recall trials, 2,511 delayed recall verbal trials, 1,314 static spatial memory trials, and 1,274 dynamic spatial memory trials (Supplementary Figures S1, S2).

Following unblinding of diagnostic categories, our analytic cohort for the verbal memory test comprised 315 participants (Supplementary Figure S1): 34 HD, 217 pwMS, subdivided into 96 relapsing-remitting (RR-MS), 61 secondary-progressive (SP-MS), and 60 primary-progressive (PP-MS), 7 clinically isolated syndrome (CIS), 2 radiologically isolated syndrome (RIS), 29 non-inflammatory neurological diseases (NIND), and 26 other inflammatory neurological diseases (OIND). Slightly lower numbers of participants contributed to the spatial memory test cohort (Supplementary Figure S2). Participant demographics are detailed for both tests in Table 1.

To develop robust, unbiased composite biomarkers via supervised machine learning, we randomly split the 217 pwMS into a training set (145 patients, two-thirds) and a validation set (72 patients, one-third). The validation cohort was held out entirely during feature selection and model building and was only used to assess final model performance.

### 3.3 Processing of NeuFun-TS verbal memory tests raw data and deriving digital outcomes

In processing the raw NeuFun-TS data, we first extracted every tap event (i.e., selection, deselection and its timestamp) from the Firebase logs for each verbal and spatial memory trial.

For the verbal task, we began by calculating each participant's percent-correct score (the number of correctly matched word pairs divided by the total attempted, times 100). To align higher values with greater cognitive impairment, this percentage was inverted (by subtracting from 100) and log₁₀-transformed, yielding our “Verbal Recall Impairment (VRI)” metric, which rises in tandem with cognitive disability.

Next, we examined test timing, which comprises IPS. In an attempt to separate sensory-motor delay from purely cognitive IPS contributions we computed the minimal inter-tap interval within each trial and named it individualized Sensory-Motor Processing Threshold (iSMPT) on the premise that it reflects the fastest response for cognitively least-demanding task. Formally, for each trial *i*, iSMPT was defined as

$$\mathrm{iSMP}T_{i}=\min(\Delta t_{i,1},\Delta t_{i,2},\ldots ,\Delta t_{i,n}),$$

where Δt denotes consecutive inter-tap intervals within the trial.

While iSMPT generally integrates sensory-motor delay due to physical disability and (the minimal) cognitive information processing delay, iSMPT can be abnormally elevated in cognitively intact individuals who have only sensory-motor delay, e.g., caused by a single cervical spinal cord lesion. Thus, we would expect that iSMPT will correlate with both physical disability outcomes and cognitive disability outcomes, capturing a global processing bottleneck rather than isolated motor output.

**Figure 2:**
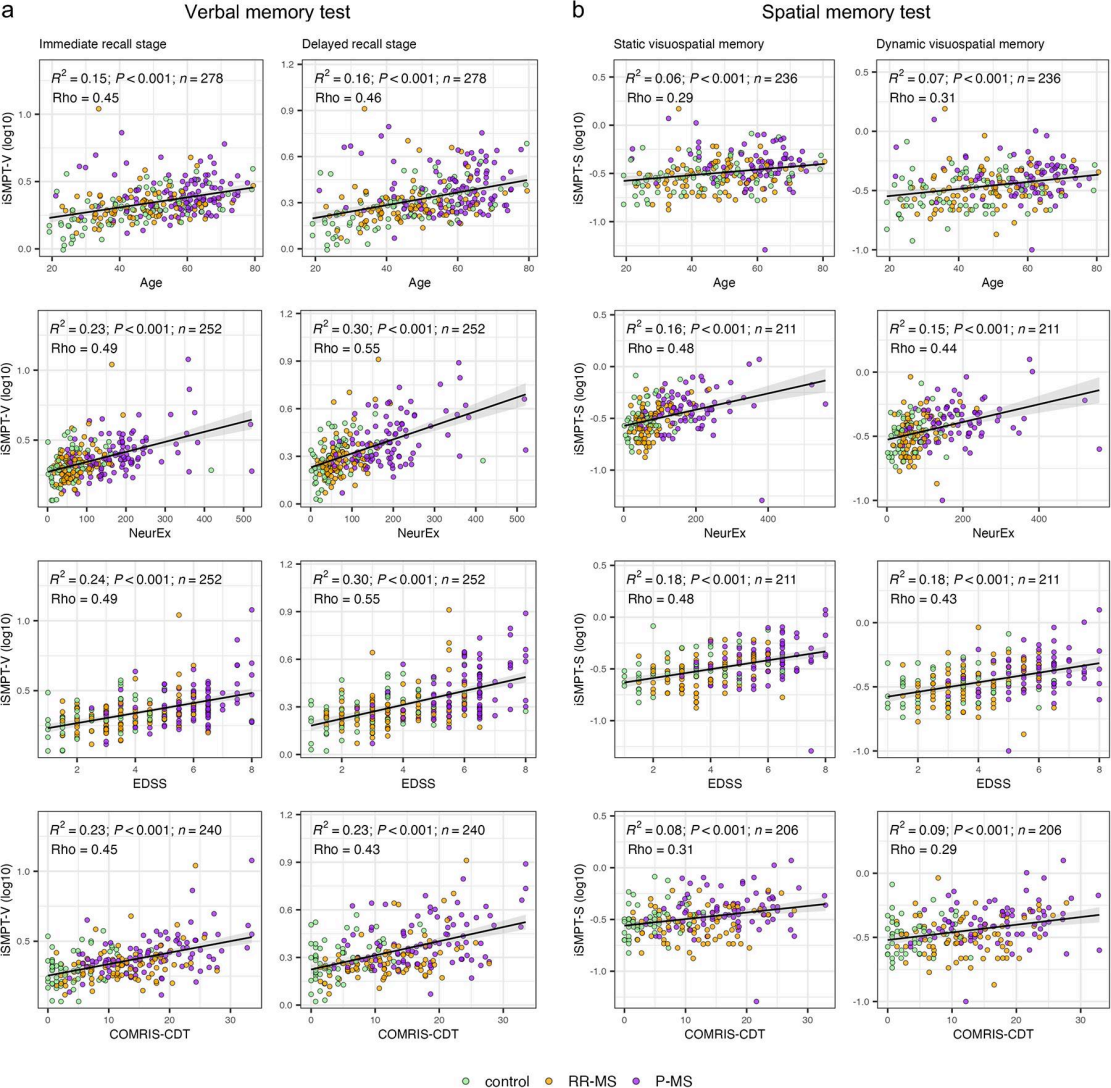
Associations between individualized sensory-motor processing threshold-verbal (iSMPT-V) and -spatial (iSMPT-S) and clinical measures across memory tests. **(a)** Verbal memory: first-trial iSMPT-V plotted against Age, NeurEx, EDSS and COMRIS-CDT for immediate recall (left) and delayed recall (right). **(b)** Spatial memory: first-trial iSMPT-S plotted against the same four predictors for the static (left) and the dynamic (right) visuospatial memory stage. Confidence intervals for all metrics are in Supplementary Table S1. Points represent controls (all non-MS patients, green), relapsing-remitting MS (RR-MS, orange) and progressive MS (P-MS, purple). Each panel reports the linear fit's coefficient of determination (R²), its *p*-value (*P*), sample size (*n*), and the Spearman correlation coefficient (Rho).

In support of this hypothesis, across all memory modules, iSMPT (derived separately from verbal memory tests: iSMPT-V [for immediate and delayed recall] and spatial memory tests: iSMPT-S [for static and dynamic test]) correlated more strongly with neurological disability scales (EDSS Rho = 0.49–0.55, R² = 0.24–0.30; NeurEx Rho = 0.49–0.55, R² = 0.23–0.30; all *P* < 0.001) than with age alone Rho = 0.45–0.46, R² = 0.15–0.16; all *P* < 0.001), confirming its sensitivity to full (i.e., physical and cognitive) disability (Figure 2, corresponding 95% confidence intervals for all Rho and R² are provided in Supplementary Table S1). iSMPT-V also correlated with brain MRI volumetric outcomes (Supplementary Figure S4a) such as BPFr (Rho = −0.46 to −0.48, R^2^ = 0.21–0.23, *P* < 0.001), ventricular volume (Rho = 0.47–0.50, R^2^ = 0.19–0.21, *P* < 0.001) and lesion volume (Rho = 0.35–0.43, R^2^ = 0.22–0.23, *P* < 0.001). However, from all MRI outcomes, the iSMPT-V correlated the strongest with the published, machine learning-derived COMRIS-CTD (Rho 0.43–0.45, R² = 0.23; *P* < 0.001; Figure 2a), which is based on semi-quantitative grading of atrophy and lesion load in different areas of CNS tissue (24). Similarly to the verbal test, iSMPT-S correlates stronger with disability and MRI measure of CNS destruction (EDSS Rho = 0.43–0.48, R² = 0.18; NeurEx Rho = 0.44–0.48, R² = 0.15–0.16; COMRIS-CTD Rho = 0.29–0.31, R² = 0.08–0.09; all *P* < 0.001) than with age alone (Rho = 0.29–0.31, R² = 0.06–0.07, *P* < 0.001; Figure 2b), supporting its interpretation as an integrated sensory-motor processing metric rather than a simple motor surrogate.

Importantly, the utility of iSMPT lies in its ability to account for sensory-motor processing constraints that disproportionately affect individuals with physical disability. By subtracting iSMPT representing a subject-specific sensory-motor processing threshold from total inter-tap latency, individuals with motor impairment but preserved cognition are expected to exhibit adjusted response-time distributions comparable to healthy controls. Any additional delay remaining after this adjustment, captured by the Recall Latencies adjusted (RLa), therefore reflects processing delays attributable specifically to increased cognitive task demands. Thus, this produced four additional verbal memory-based biomarkers: Immediate- (ImmVRI) and Delayed Verbal Recall Impairment (DelVRI) and Immediate- (ImmVRLa) and Delayed Verbal Recall Latency adjusted (DelVRLa).

### 3.4 Verbal memory digital biomarkers correlate weakly to moderately with cognitive subscores of clinician-derived disability tests and with imaging biomarkers of brain damage

**Figure 3:**
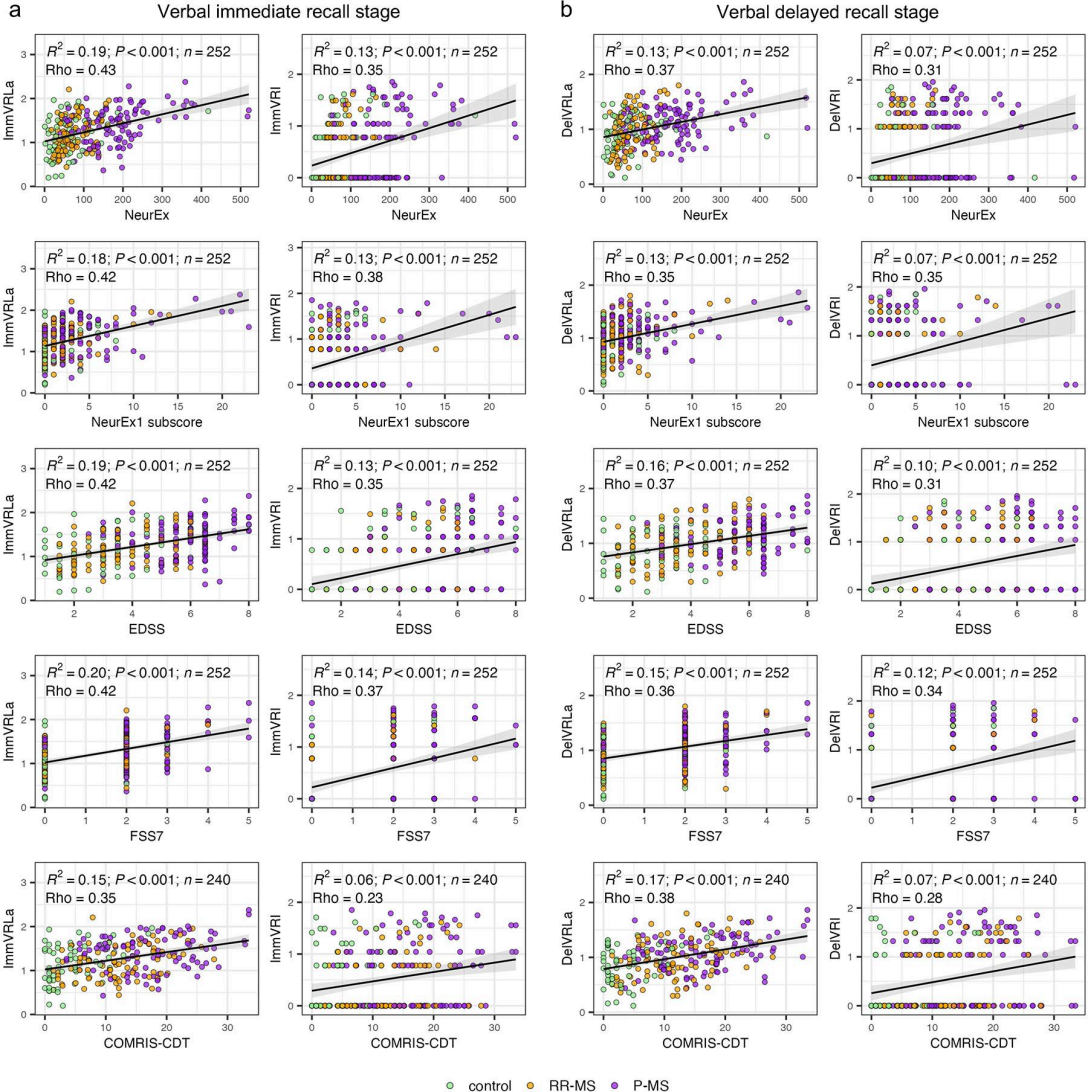
Associations between verbal recall latency adjusted [Immediate (ImmVRLa) and delayed (DelVRLa)] and verbal recall impairment [Immediate (ImmVRI) and delayed (DelVRI)] with clinical and MRI measures across two stages of the verbal memory test. **(a)** Verbal memory immediate recall: first-trial ImmVRLa (left) and ImmVRI (right) plotted against NeurEx, cognitive subscore of Neurex (Neurex1 subscore), EDSS, Kurtzke Functional System Score 7 (FSS7), and COMRIS-CDT. **(b)** Verbal memory delayed recall: first-trial DelVRLa (left) and DelVRI (right) plotted against the same five clinical and MRI outcomes. Confidence intervals for all metrics are in Supplementary Table S2. Points represent controls (all non-MS patients, green), relapsing-remitting MS (RR-MS, orange) and progressive MS (P-MS, purple). Each panel reports the linear fit's coefficient of determination (R²), its *p*-value (*P*), sample size (*n*), and the Spearman correlation coefficient (Rho).

To evaluate the validity of verbal memory biomarkers adjusted for iSMPT, we first examined how each metric related to both cognitive subdomains (Kurtzke FSS7, NeurEx1 cognitive subscores) and global disability scales (EDSS, and overall NeurEx) derived from clinician-administered exams. As shown in Figure 3, all four digital outcomes demonstrated statistically significant associations with each of these clinical measures. Notably, the immediate recall metrics yielded slightly stronger correlations than their delayed counterparts. Additionally, time-based outcomes (ImmVRLa and DelVRLa) consistently outperformed accuracy-based outcomes (ImmVRI and DelVRI) in every comparison, consistent with predominant subcortical/deep WM brain pathology in MS. Moreover, the strength of these associations with the cognitive subscores (e.g., FSS7, NeurEx1 subscore) was comparable to that with EDSS and total NeurEx. This suggests that verbal memory biomarkers capture broad neurological impairment rather than purely domain-specific deficits or, that cognitive and physical disabilities advance generally in parallel with MS evolution.

We then turned to neuroimaging correlates, assessing each verbal memory biomarker against volumetric MRI measures (Supplementary Figure S5) and the COMRIS-CTD (Figure 3, bottom row). The BPFr displayed modest correlations for ImmVRLa/DelVRLa (Rho = −0.06 to −0.19, R² = 0.06–0.12, *P* < 0.008), as did brain ventricular volume (Rho = 0.22–0.40, R² = 0.04–0.12, *P* < 0.02) and T2 lesion volume (Rho = 0.28, R² = 0.07–0.11, *P* < 0.003). Like we observed for iSMPT-V, from all imaging outcomes COMRIS-CTD correlated strongest with remaining verbal memory biomarkers: ImmVRLa/DelVRLa (Rho = 0.35–0.38, R² = 0.15–0.17, *P* < 0.001), extending even to the ImmVRI/DelVRI predictors [Rho = 0.23–0.28, R² = 0.06–0.07, *P* < 0.001, which showed marginal or no correlations with fully quantitative MRI outcomes (Supplementary Figure S5b)]. Nevertheless, none of the verbal-specific digital biomarkers outperformed the iSMPT-V (Figure 2a) in predicting either clinical disability or imaging-based CNS damage.

We conclude that even when adjusting for sensory-motor disability, verbal memory biomarkers correlate moderately with global disability scales and imaging biomarkers of CNS tissue destruction, but do not demonstrate preferential specificity for cognitive disability in pwMS.

### 3.5 Processing of NeuFun-TS spatial memory tests raw data and deriving digital outcomes

The spatial memory test was processed using an analogous framework, with task-specific adaptations reflecting its static and dynamic visuospatial demands. Each participant began with a raw score equal to the sum of correctly selected squares across five increasing-difficulty patterns (i.e., 4, 5, 6, 7, and 8 squares) yielding a maximum of 30 points in the static stage and, when double-weighted, 60 points in the dynamic stage. However, this “all-or-nothing” scoring obscured the fact that two subjects who both “fail” a pattern may nevertheless perform quite differently: one might get zero squares right, while another might select all but one correctly. To capture that nuance, we implemented a partial-credit algorithm for the failed level in each stage.

Concretely, suppose a participant flawlessly completes patterns 4 and 5, then struggles on pattern 6. During static testing, each new attempt at pattern 6 presents a fresh random configuration of six squares until mastery. We record the number of incorrect taps on each attempt—say, three mistakes on the first try and one on the second. We compute the average error for that level [here, (3 + 1)/2 = 2] and subtract it from the level's square count (6–2 = 4). We then add those four partial-credit tiles to the 4 + 5 already earned, giving a refined static score of 13. Dynamic scoring follows the same principle, except that each level's tile count is double-weighted [so pattern 6 carries 12 points, and if the average mistakes were 2, the partial credit would be 12—(2 × 2) = 8, and so on].

After summing these adjusted square counts, we inverted the total (so that higher values correspond to greater impairment) and applied a log₁₀ transformation to normalize the distribution, yielding Static Spatial Recall Impairment (StaticSRI) and Dynamic Spatial Recall Impairment (DynamicSRI). This approach preserves the hierarchical structure of task difficulty while providing a continuous, high-resolution metric that reflects both outright success and near-miss performance on challenging patterns.

We also captured every self-corrected error (i.e., instances where an incorrect square was tapped then deselected) as a mistake count per stage, log₁₀-transformed for normality, yielding Static Spatial Recall Mistakes (StaticSRM) and Dynamic Spatial Recall Mistakes (DynamicSRM).

Finally, mirroring the verbal workflow, we generated individualized Sensory-Motor Processing Threshold(s)-Spatial (iSMPT-S), Static Spatial Recall Latency adjusted (StaticSRLa) and Dynamic Spatial Recall Latency adjusted (DynamicSRLa).

We further examined the ability of iSMPT to dissociate motor-related processing delay from cognitive slowing by stratifying participants into four quadrants based on the joint distribution of iSMPT-S and adjusted recall latency (StaticSRLa and DynamicSRLa). This approach separates individuals with combined severe physical and cognitive impairment (Q1), minimal physical but severe cognitive impairment (Q2), minimal physical and cognitive impairment (Q3), and severe physical but relatively preserved cognitive function (Q4).

Consistent with this interpretation, participants classified in Q4 characterized by elevated iSMPT-S but low adjusted recall latency exhibited significantly greater spinal cord tissue damage alongside significantly lower supratentorial brain involvement compared with cognitively impaired groups (Supplementary Figure S6). In contrast, participants with high adjusted recall latency but relatively preserved iSMPT-S (Q2) demonstrated greater supratentorial pathology with less spinal cord involvement. These findings support the role of iSMPT as a marker of sensory-motor processing load, while adjusted recall latencies selectively capture cognitive slowing.

### 3.6 Spatial memory digital biomarkers correlate stronger with clinician-derived cognitive disability subscores than verbal memory biomarkers

**Figure 4:**
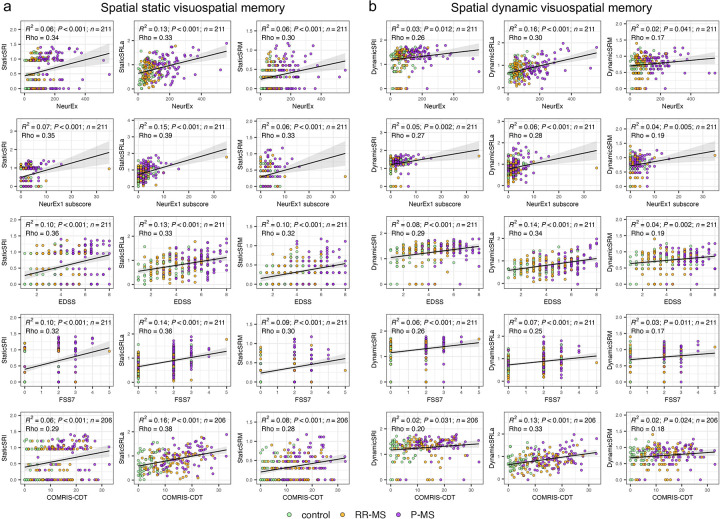
Associations between spatial recall impairment (SRI), spatial recall latency adjusted (SRLa) and spatial recall mistakes (SRM) with clinical and MRI measures across two stages of the spatial memory test. **(a)** Static visuospatial memory stage: first-trial StaticSRI (left), StaticSRLa (middle) and StaticSRM (right) plotted against NeurEx, cognitive subscore of Neurex (Neurex1 subscore), EDSS, Kurtzke Functional System Score 7 (FSS7), and COMRIS-CDT. **(b)** Dynamic visuospatial memory stage: first-trial DynamicSRI (left), DynamicSRLa (middle) and DynamicSRM (right) plotted against the same five clinical and MRI outcomes. Points represent controls (all non-MS patients, green), relapsing-remitting MS (RR-MS, orange) and progressive MS (P-MS, purple). Each panel reports the linear fit's coefficient of determination (R²), its *p*-value (*P*), sample size (*n*), and the Spearman correlation coefficient (Rho). Confidence intervals for all metrics are in Supplementary Table S3.

Next, we assessed how each spatial memory metric related to clinician-derived cognitive scores and the COMRIS-CTD imaging outcome (Figure 4). When we correlated static stage outcomes (StaticSRI, StaticSRM and especially StaticSRLa) against the Kurtzke FSS7 cognitive subscore, we observed Spearman Rho values of 0.30–0.36 and R² values of 0.09–0.14 (*P* < 0.001), while the dynamic stage counterparts still achieved significant albeit weaker correlations (Rho = 0.17–0.26, R² = 0.03–0.07; *P* < 0.011). Similar patterns emerged for the NeurEx1 cognitive subscore (static: Rho = 0.33–0.39, R² = 0.06–0.15, *P* < 0.001; dynamic: Rho = 0.19–0.28, R² = 0.04–0.06, *P* < 0.005).

Crucially, correlations of StaticSRLa with cognitive subscores of neurological examination (NeurEx1 and FSS7) outperformed its correlation with global disability outcomes (NeurEx and EDSS) supporting our hypothesis that subtracting iSMPT-S from each test answer better isolates pure cognitive slowing. Once again, time-based SRLa emerged as the top performer across both spatial stages, yielding the strongest associations not only with FSS7 and NeurEx1 subscores but also with the COMRIS-CTD.

### 3.7 Development and validation of composite digital biomarkers (ordinal logistic regression approach)

Our prior work with NeuFun-TS has shown that aggregating multiple digital biomarkers into a single score can amplify clinical signal. The workflow has two obligatory steps. Step 1 (model derivation): digital biomarkers from the training cohort are combined so that the resulting score best aligns with a clinician-defined reference. Step 2 (model validation): the derived equation is frozen and applied to an independent validation cohort that played no part in model building. Only this second step yields an unbiased estimate of real-world performance.

For most NeuFun-TS modules the reference outcome was continuous, allowing straightforward linear regression. Cognitive subscores of the neurological exam, however, are ordinal and far from linear. We therefore reframed the problem: instead of predicting a numeric subscore, we asked whether memory performance could separate three diagnostic categories that, by clinical consensus, lie on an ascending cognitive impairment continuum: HD < RR-MS < progressive MS (P-MS, which includes SP-MS and PP-MS). Ordinal logistic regression, specifically the proportional-odds model, accommodates such ranked outcomes and returns the optimal weight for each digital biomarker.

**Figure 5:**
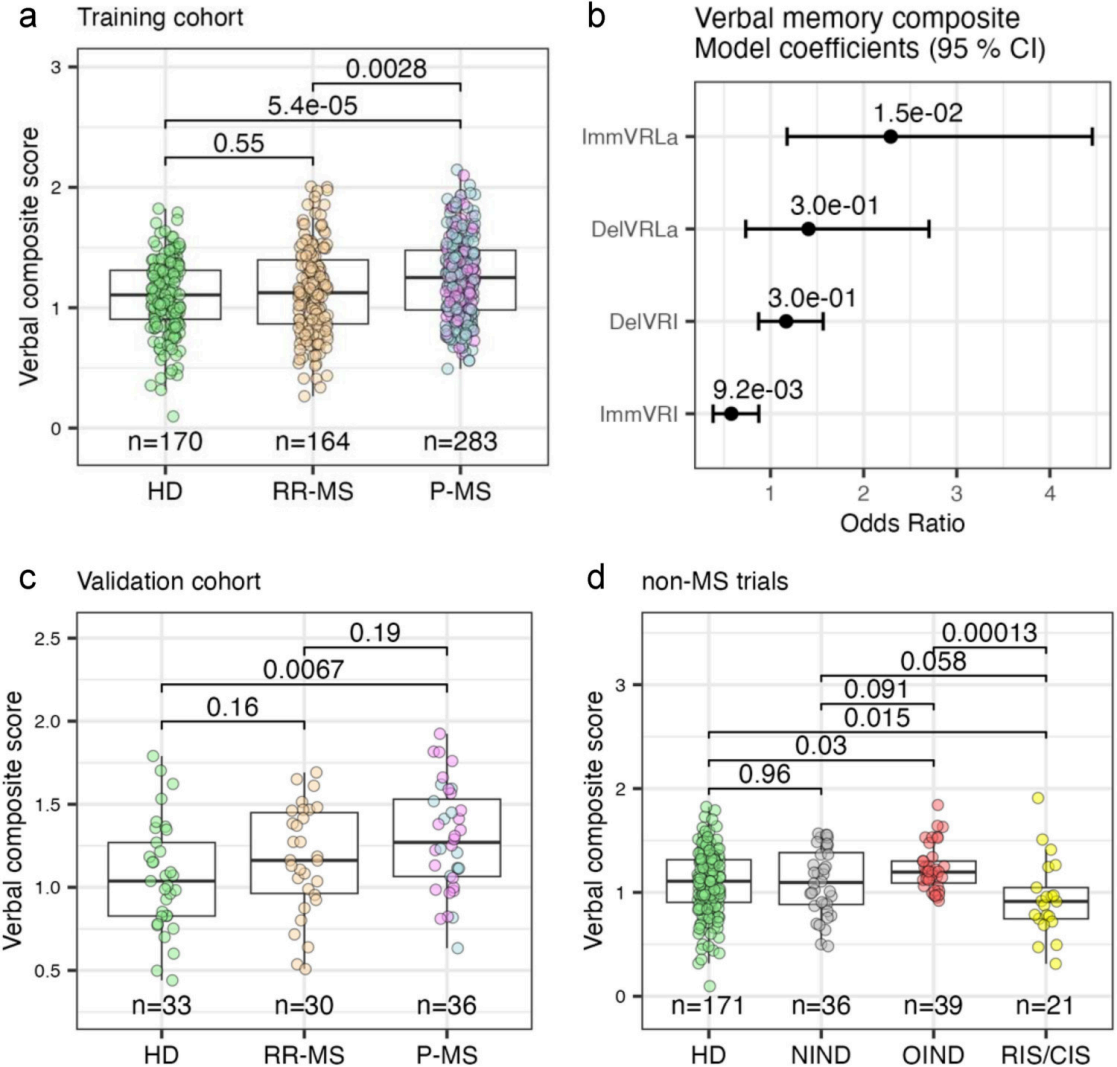
Verbal memory composite score distributions and model coefficients. **(a)** Training cohort: boxplots of composite scores over up to 10 consecutive trials per subject, for healthy donors (HD), relapsing-remitting MS (RR-MS) and progressive MS (P-MS); sample sizes are shown below each group. Pair-wise Wilcoxon signed-rank *p*-values are indicated. **(b)** Ordinal logistic regression model: odds ratios (points) and 95% confidence intervals (bars) for four digital predictors—Immediate Verbal Recall Latency adjusted (ImmVRLa), Delayed Verbal Recall Latency adjusted (DelVRLa), Delayed Verbal Recall Impairment (DelVRI) and Immediate Verbal Recall Impairment (ImmVRI)—on the verbal composite score. **(c)** Validation cohort: composite score distribution from the first trial per subject, across HD, RR-MS and P-MS. Wilcoxon rank-sum *p*-values are indicated. **(d)** Non-MS samples: composite score distribution in HD, non-inflammatory neurological disease (NIND), other inflammatory neurological disease (OIND) and radiologically/clinically isolated syndrome (RIS/CIS); Wilcoxon signed-rank *p*-values are indicated. *All available HD trials were used to build the model and then reused in both the validation and non-MS cohorts.* Confidence intervals for all metrics are in Supplementary Table S4.

First, we generated verbal memory composite score. To maximize training data we used up to the first ten trials per subject, yielding 617 immediate recall recordings. Ten-fold cross-validation tuned the model (Figure 5). An independent validation set of 99 participants contributed only one (their first) test. The verbal composite distinguished P-MS from HD with statistical significance but failed to separate RR-MS from either HD or P-MS and showed no power to differentiate HD from NIND controls, and marginal significance to do so in OIND controls.

**Figure 6:**
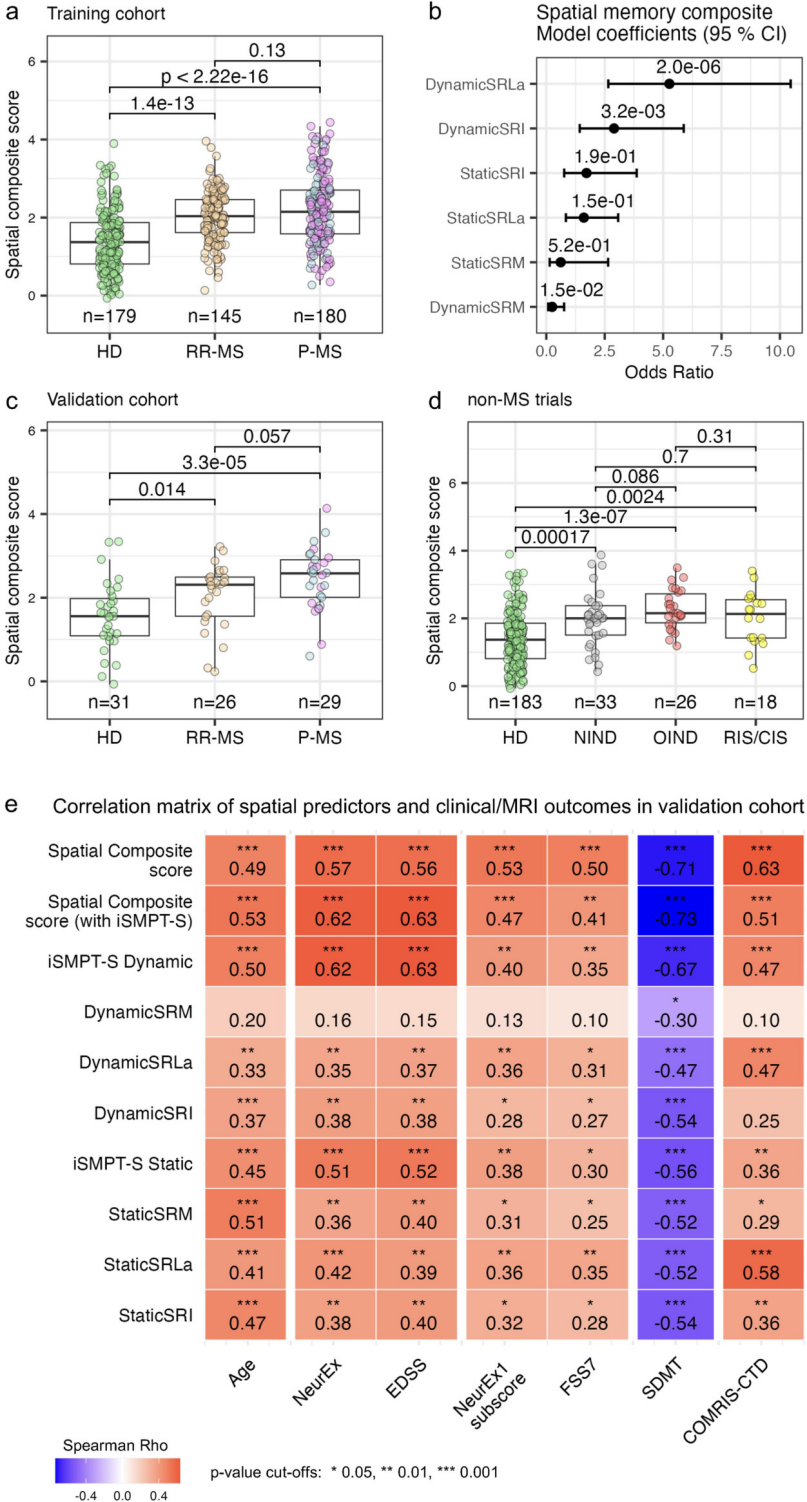
Spatial memory composite score distributions and model coefficients. **(a)** Training cohort: boxplots of composite scores over up to 10 consecutive trials per subject, for healthy donors (HD), relapsing-remitting MS (RR-MS) and progressive MS (P-MS); sample sizes are shown below each group. Wilcoxon rank-sum *p*-values are indicated. **(b)** Ordinal logistic regression model: odds ratios (points) and 95% confidence intervals (bars) for six digital predictors—Static Spatial Recall Latency adjusted (StaticSRLa), Dynamic Spatial Recall Latency adjusted (DynamicSRLa), Static Spatial Recall Impairment (StaticSRI), Dynamic Spatial Recall Impairment (DynamicSRI), Static Spatial Recall Mistakes (StaticSRM) and Dynamic Spatial Recall Mistakes (DynamicSRM)—on the spatial composite score. **(c)** Validation cohort: composite score distribution from the first trial per subject, across HD, RR-MS and P-MS. Pair-wise Wilcoxon signed-rank *p*-values are indicated. **(d)** Non-MS samples: composite score distribution in HD, non-inflammatory neurological disease (NIND), other inflammatory neurological disease (OIND) and radiologically/clinically isolated syndrome (RIS/CIS); Pair-wise Wilcoxon rank-sum *p*-values are indicated. *All available HD trials were used to build the model and then reused in both the validation and non-MS cohorts.* **(e)** Spearman correlation matrix between all spatial memory digital biomarkers [Static/Dynamic individualized Sensory-Moror Processing Threshold-Spatial (iSMPT-S), Static/DynamicSRLa, Static/DynamicSRI, Static/DynamicSRM] and clinical/MRI outcomes in the validation cohort (first trial per subject). Each cell shows the Spearman correlation coefficient and significance stars (*/**/*** for *P* < 0.05/0.01/0.001). Confidence intervals for all metrics are in Supplementary Table S5.

Applying the same pipeline to the spatial module produced a markedly stronger tool (Figures 6a–d). The composite separated 31 HD from 26 RR-MS, 29 P-MS, 33 NIND, 26 OIND, and even 18 RIS/CIS participants (all *P* < 0.01), although given the small sample size of the RIS/CIS group, these comparisons should be interpreted as exploratory. No single spatial biomarker matched this performance (Supplementary Figure S7a), a finding that held up even when up to ten trials per subject were analyzed (Supplementary Figure S7b).

Because iSMPT-S captures sensory-motor speed, we built sensitivity models that appended iSMPT-S to the spatial feature set (Supplementary Figure S8). As expected, iSMPT-S received the heaviest weight and the resulting composite achieved the largest effect sizes across all diagnostic contrasts, albeit at the cost of reduced cognitive specificity. The final coefficients and odds ratios for the ordinal logistic regression models underlying the spatial memory composite with and without iSMPT predictors are provided in Supplementary Table S12. Confusion matrices for all 4 logistic regression models and their prediction accuracies in the independent validation cohort are in Supplementary Table S13.

We next combined all verbal and spatial inputs into a “global memory” model (Supplementary Figure S9). Spatial memory markers dominated the weighting scheme, and the hybrid score offered no advantage over the spatial memory-only composite. Thus, the verbal test contributed no added value over spatial memory test alone.

Although we built the spatial memory composites to separate diagnostic groups, a valid composite should also eclipse each parent biomarker when correlated with continuous measures of disease burden. That expectation was fulfilled (Figure 6e). The spatial composite version with iSMPT-S (as well as iSMPT-S alone) displayed the tightest links to global disability, correlating with EDSS and total NeurEx at Rho = 0.62–0.63 (*P* < 0.001). Conversely, the composite without iSMPT-S, which isolates cognitive latency, proved most sensitive to cognitive subscores (FSS7 and NeurEx1 subscore, Rho = 0.50–0.53, *P* < 0.001) and to the COMRIS-CTD metric of tissue destruction (Rho = 0.63, *P* < 0.001). These results strongly support our hypothesis that we can (at least partially) separate slowing of sensory-motor processing from slowing of cognitive functions that underlie static and dynamic non-verbal memory.

We conclude that the spatial memory composites, whether configured for cognitive specificity or overall neurological burden, outperform every individual memory digital biomarker and constitute the most clinically informative digital measures we have developed.

### 3.8 Spatial memory test can be used to quality control smartphone SDMT outcome and provides reasonable reproducibility for longitudinal testing

A core design principle of NeuFun-TS is that each neurological domain is assessed by more than one task, allowing independent tests to serve as internal validity checks. SDMT reflects either IPS alone or IPS combined with working memory, depending on participant's strategy. As the symbol-digit key remains visible throughout the test, participants may choose to rely on working memory rather than visual lookup, potentially achieving faster performance at the cost of increased errors.

We therefore hypothesized that spatial memory performance, which captures both IPS and visuospatial processing/working memory, should reliably predict SDMT scores, because both tests measure overlapping neuronal functions. If this prediction holds true, large discrepancies between the predicted and observed SDMT values could be used to flag unreliable trials in real time.

**Figure 7:**
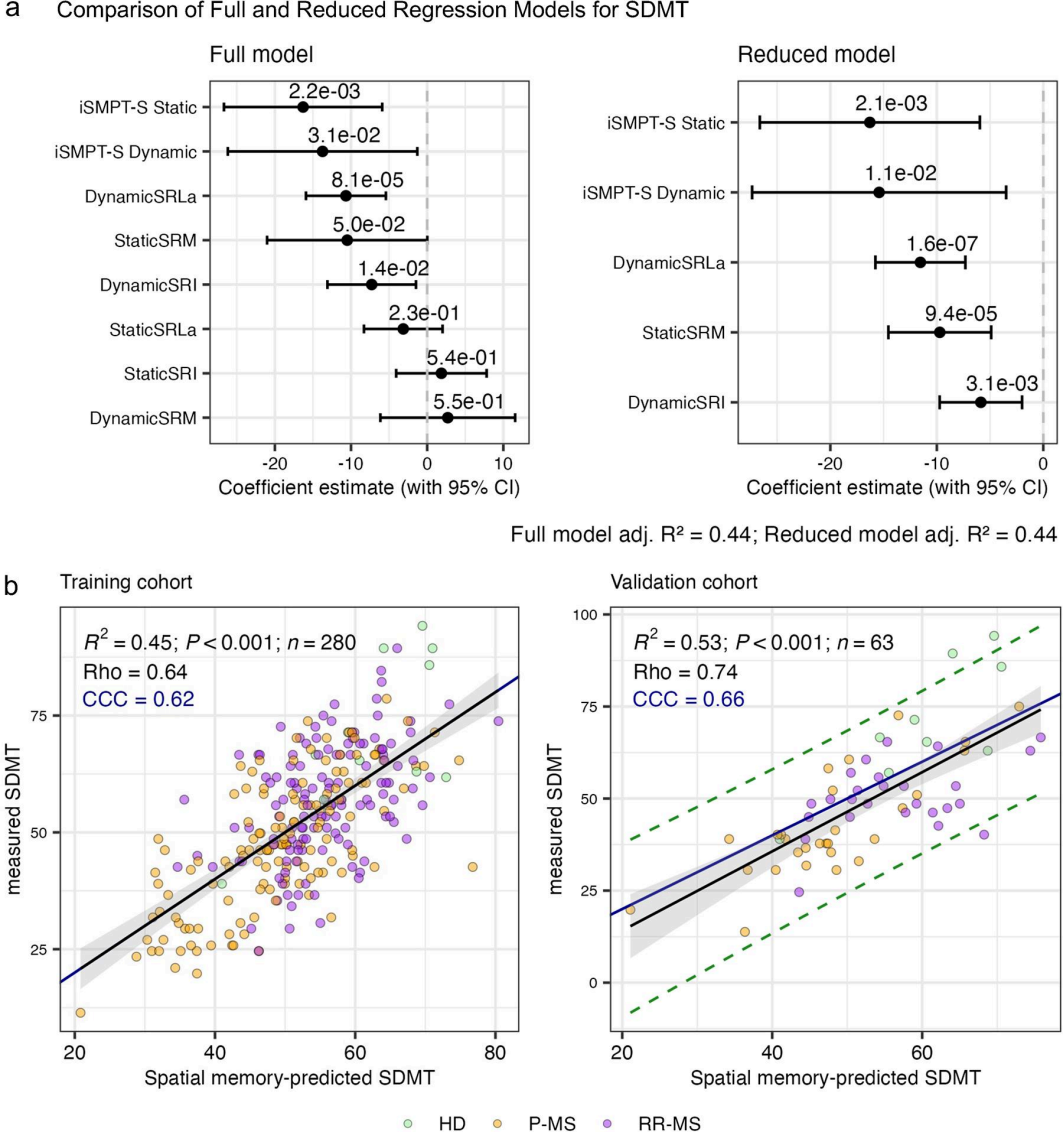
Spatial memory biomarkers predict symbol digit modalities test (SDMT). **(a)** Regression coefficients for eight spatial memory predictors in the full model (left) and the five variable reduced model (right). Predictors include Spatial Recall Latency adjusted (Static/DynamicSRLa), Spatial Recall Impairment (Static/DynamicSRI), Spatial Recall Mistakes (Static/DynamicSRM) and individualized Sensory-Motor Processing Threshold-Spatial (iSMPT-S Static/Dynamic). Black dots mark coefficient estimates and whiskers show 95% confidence intervals (CI); the reduced model retains only the five predictors that significantly contribute to SDMT prediction. **(b)** Observed versus predicted SDMT in the training cohort (left; up to 10 trials per subject) and the validation cohort (right; first trial per subject). Points are colored by diagnosis: healthy donors (HD, green), relapsing-remitting MS (RR-MS, orange) and progressive MS (P-MS, purple). The coefficient of determination (R²), its *p*-value (*P*), sample size (*n*), the Spearman Rho, and the concordance correlation coefficient (CCC) are displayed on top of the plots. The black line and gray band show the linear regression fit ± 95% CI, the dark blue line is the identity (1:1) line; in the validation panel the green dashed lines mark the 95% prediction interval. Confidence intervals for all metrics are in Supplementary Table S6.

To test this hypothesis, we built a stepwise multiple linear regression model in the training cohort, using all spatial memory biomarkers, including iSMPT-S, as candidate predictors (Figure 7a). The resulting equation explained 53% (CI: 38%–67%) of SDMT variance in an independent validation set of 63 participants. In that unseen cohort the model achieved a Spearman correlation of *Rho* = 0.7*4*, and a Lin's concordance correlation coefficient (CCC) of 0.*66* (*P* < 2.2 × 10^−16^), indicating accurate 1:1 agreement between predicted and observed scores (Figure 7b).

Because residuals in the validation cohort were approximately normal and homoscedastic, we computed a 95% prediction interval around the regression line (Figure 7b, right panel). This interval defines the expected range of SDMT values given an individual's spatial memory performance.

During unsupervised NeuFun-TS monitoring, observed SDMT scores falling outside this prediction interval (conditional on spatial memory performance) are automatically flagged as potentially unreliable. Such deviations may arise from non-cognitive factors, including distraction, fatigue, misunderstanding of instructions, or technical issues. Flagged trials can prompt immediate test repetition, exclusion from longitudinal averaging, or cautious interpretation by clinicians. In this manner, the prediction envelope functions as an internal, data-driven quality-control mechanism that enhances the reliability of unsupervised cognitive monitoring without external supervision.

### 3.9 Spatial memory composite has good reliability in longitudinal testing, but like SDMT, its sensitivity to identify memory decline on individual level would increase with implementation of period averages in granular testing

**Figure 8:**
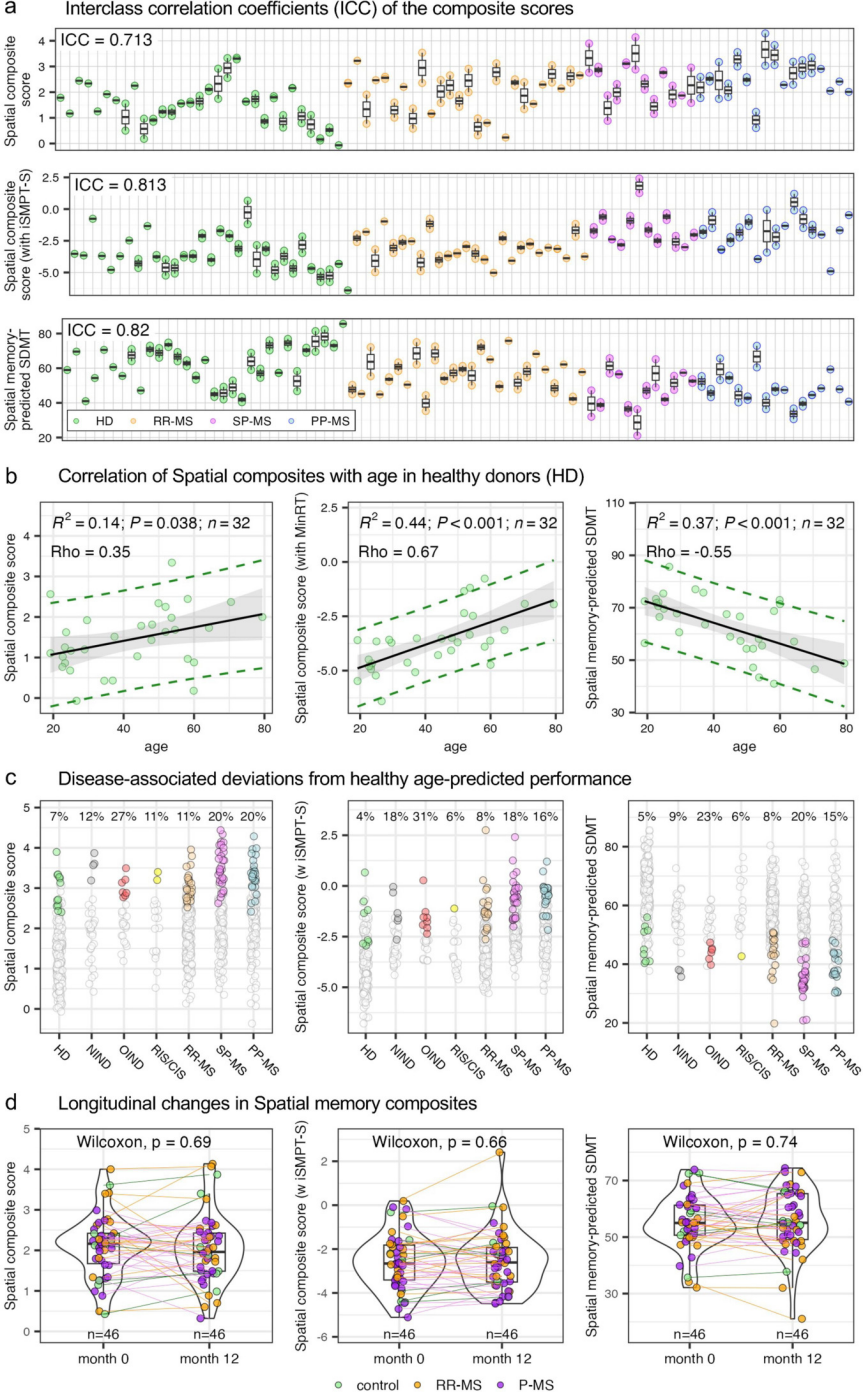
Reliability and longitudinal change in spatial composites. **(a)** Test–retest reproducibility across two visits: each small box + dots represent one subject's repeated measurements of (top) the spatial composite score, (middle) the spatial composite score with individualized Sensory-Motor Processing Threshold-Spatial (iSMPT-S), and (bottom) spatial memory-predicted Symbol Digit Modalities Test (SDMT). Colors denote diagnostic group: healthy donors (HD, green), relapsing–remitting MS (RR-MS, orange), secondary-progressive MS (SP-MS, purple) and primary-progressive MS (PP-MS, blue) and the intraclass correlation coefficient (ICC) for each metric is shown. **(b)** Cross-sectional associations with age in the HD cohort—each dot represents a median of measured points across one year: scatterplots of each metric versus age, with a linear regression fit (black line ± 95% CI). The coefficient of determination (R²), its *p*-value (*P*), sample size (*n*), and the Spearman Rho are displayed in the plots. The green dashed lines mark the 90% prediction interval. **(c)** Disease-associated deviations from healthy age-predicted performance: First 10 trials per subject are plotted for three metrics: (left) spatial composite score, (center) spatial composite score adjusted for iSMPT-S, and (right) spatial memory–predicted SDMT. Gray circles mark trials whose values lie within the 90% prediction interval derived from HD; colored circles denote trials that fall outside this interval, by diagnosis. The percentages above each diagnostic group represent proportion of trials above HD 90% prediction interval for corresponding age. NIND—non-inflammatory neurological disease, OIND—other inflammatory neurological disease, RIS/CIS—radiologically/clinically isolated syndrome. **(d)** Paired 12-month changes: violin plots of each metric at baseline (month 0) versus month 12, with individual subject trajectories, sample size (*n*) and Wilcoxon signed-rank test *p*-values. Confidence intervals for all metrics are in Supplementary Table S7.

Although the spatial memory composite discriminates well between diagnostic groups, its clinical utility ultimately depends on how reliably it tracks change within the same individual over time. We therefore examined longitudinal reproducibility in the independent validation cohort, in which each participant completed two trials separated by at least one day. Reliability was quantified with the ICC, which compares within-subject variance to between-subject variance on a 0–1 scale (values of 0.50–0.75 are considered “moderate,” 0.75–0.90 “good,” and >0.90 “excellent”). The composite that included iSMPT-S achieved an ICC of 0.813 (CI: 0.689–0.884), while the cognitively-specific version without iSMPT-S reached 0.713 (CI: 0.606–0.789). A third metric, the spatial memory model that predicts SDMT, showed an ICC of 0.82 (Figure 8a). Taken together, these results place the two iSMPT-containing scores firmly in the “good” range and the memory-only composite near the upper edge of “moderate” reliability.

Because cognitive speed and accuracy decline with normal aging, we next asked whether spatial memory performance exhibited an age effect in HD. All three composites correlated with HD age (Figure 8b), reinforcing the need to judge individual results against an age-adjusted 90% prediction interval derived from the healthy cohort (Figure 8c). Such normative curves allow us to distinguish disease-related decline from changes that merely reflect advancing age.

To probe disease-associated progression, we identified pwMS who completed the spatial memory test at baseline and again after 12 months. A paired Wilcoxon signed-rank test revealed no group-level difference between month 0 and month 12 (Figure 8d), suggesting that detectable cognitive decline over a single year is uncommon in this sample, or that the signal is masked by week-to-week variability. Indeed, home testing in Cohort 3 displayed the same modest day-to-day fluctuations (Supplementary Figure S10) we previously documented for the smartphone SDMT (15). Such short-term fluctuations almost certainly reflect non-cognitive influences (e.g., fatigue, sleep quality, motivation, alcohol or drug use, concurrent medications or performance noise) that are inherent to daily cognitive performance even in healthy subjects.

To formally assess how repeated sampling improves sensitivity to individual cognitive change, we analyzed a subset of 10 participants who completed dense longitudinal spatial memory testing. Visual inspection of individual trajectories revealed substantial day-to-day variability in single-trial measurements, despite relatively stable or slowly evolving long-term trends.

We therefore quantified within-subject variance before and after temporal averaging of consecutive trials. Averaging four consecutive trials reduced within-subject variance by a median of 79.5%, with consistent variance reduction observed across all individuals (Supplementary Figure S10). This reduction reflects suppression of short-term, non-cognitive fluctuations while preserving underlying longitudinal trajectories.

These findings demonstrate that dense, repeated sampling combined with short-window averaging substantially improves the signal-to-noise ratio of smartphone-based cognitive measures, thereby enhancing sensitivity to individual-level cognitive change even when overt progression is not detectable over short follow-up intervals.

## 4 Discussion

Identifying and quantifying subtle cognitive decline associated with physiological aging or progressive CNS disease remains a significant challenge. The current gold standard involves the supervised administration of standardized batteries of cognitive tests, scored by trained professionals. However, in broad clinical practice, these batteries are typically administered infrequently (e.g., once every few years) and generally only after a specialist's referral for suspected cognitive impairment.

We believe that advances in digital technologies can both augment and democratize healthcare by providing tools that can reliably measure individual trajectories of cognitive and physical neurological functions in an unsupervised manner, all within the convenience of the subjects' homes. NeuFun-TS is one such emerging digital tool among many others currently being developed (31–35). A comprehensive review of these emerging technologies is beyond the scope of this paper; we refer the reader to excellent review publications on this topic (2, 36–39).

From a clinical and regulatory perspective, NeuFun-TS is a self-administered, smartphone-based assessment designed to screen for varied neurological dysfunctions. It aims to identify subtle deficits or abnormal longitudinal trajectories, providing data that must be confirmed by an appropriate specialist. Its intended role is to complement established neurological examinations, neuropsychological testing, and imaging by providing high-frequency, patient-generated data. The platform aligns with emerging frameworks for Software as a Medical Device, particularly for low- to moderate-risk monitoring and clinical decision-support tools. NeuFun-TS employs transparent, interpretable models and clinically familiar outputs that allow results to be readily understood and traced to their underlying data. The platform does not automate clinical decisions but provides quantitative information to support clinician judgment. Its unsupervised and scalable design further enables deployment in routine care and decentralized clinical trials.

To our knowledge, the key differentiating design features of NeuFun-TS are: 1. Data-driven iterative design: guided by criterion validity, reliability, and sensitivity/specificity against the gold-standard components of a comprehensive neurological examination (i.e., NeurEx) and parallel brain imaging performed concurrently with the smartphone-based tests. 2. Independent cohort validation and effect size assessments. 3. Internal quality assessments of the reliability of the results by assessing overlapping neurological domains using independent tests.

Our results confirm that most prominent cognitive impairment in MS is IPS slowing, reflected by all digital biomarkers that measure reaction times: iSMPT and adjusted latencies outcomes from all 4 stages of the memory tests. As SDMT also reflects IPS slowing, it is not surprising that, using machine-learning, we developed composite outcome that retained strong SDMT correlation in the validation cohort. The single strongest predictor was iSMPT-S, whose tight association with global disability and COMRIS-CTD suggests that it captures not only cognitive latency but also the sensory-motor execution needed to translate a decision into action. Far from diminishing iSMPT value, our decision to subtract it from total trial duration (creating adjusted latencies) underscores its clinical relevance: in daily life, tasks such as driving depend on precisely this rapid sensory-motor-cognitive integration.

Combining multiple digital features into a single composite enhances clinical signal in two ways. First, when the underlying variables tap partially independent domains, aggregation increases their correlation with global outcomes. Second, when the variables overlap, aggregation reduces random noise and improves test-retest reliability. Nonetheless, the verbal memory composite added little value over its spatial counterpart. It failed to distinguish HD from RR-MS or to separate RR-MS from P-MS, suggesting a limited dynamic range, possibly because the task was not sufficiently challenging. By contrast, the spatial memory paradigm, which incrementally increases complexity and includes a demanding dynamic stage, displayed an excellent performance range: consistent with the literature (19) the most difficult pattern (eight squares in sequence) was mastered only by the cognitively fittest participants, yet the adaptive structure prevented frustration in those with cognitive deficits. The test is also faster to complete, an important practical advantage when balancing NeuFun-TS's overall burden. For these reasons, we will retire the verbal memory module from the NeuFun-TS.

Our study has limitations. We lacked formal neuropsychological batteries, chiefly because institutional capacity is scarce and the complete neurological examination, NeuFun-TS testing, and MRI already occupy the full clinic visit. Incorporation of standardized batteries such as MACFIMS or BICAMS, which require 45–90 min of supervised administration, was therefore not feasible without compromising recruitment or longitudinal adherence. Consequently, we relied on cognitive testing embedded within the standard exam. Although sparse, cognitive subscores of EDSS and NeurEx still correlated with digital biomarkers and, critically, these cognitive measures were not used to train the machine-learning models; nevertheless, the resulting composites consistently outperformed their individual component features when evaluated against these independent clinical validators, bolstering confidence in our approach.

A second limitation concerns MRI. Policy and budget constraints curtailed volumetric acquisitions after 2021. Reassuringly, for the subset with complete imaging we found that cognitive digital biomarkers aligned most strongly with COMRIS-CTD, a semi-quantitative metric developed on an entirely separate cohort and locked in the database before this project began. This further reduces the risk of circular validation and supports the robustness of the imaging anchor, even in the absence of new volumetric data.

In addition, certain diagnostic subgroups (particularly CIS/RIS) were represented by small sample sizes. While group-level differences were observed, analyses involving these subgroups should be interpreted as exploratory, and definitive conclusions regarding early disease stages will require validation in larger, dedicated cohorts.

Finally, our longitudinal data were limited. We did not observe progression of cognitive disability in our cohort of pwMS (mostly on stable therapy) over 1 year. This is consistent with MS clinical trials, where untreated pwMS experience greater SDMT improvements when initiating active treatment, while placebo arms *improve* less (9, 10, 40). SDMT *improvements* in placebo arms are attributed to “practice effects”, which we minimized by assuring complete randomization of NeuFun-TS cognitive tests. Whether NeuFun-TS digital biomarkers can measure cognitive decline over longer time must be assessed in future studies. Based on our experience with digital SDMT (15), we expect that measuring cognitive decline on patient level requires both longer follow-up and establishing more accurate interval data by averaging granular testing (e.g., 4 weekly tests). To this end, we continue to acquire NeuFun-TS biomarkers in clinical protocols.

In summary, NeuFun-TS now contains two validated cognitive tools: the SDMT and the spatial memory test that take less than 10 min to perform and can cross-check one another to ensure data integrity during unsupervised use. The novel spatial memory test is convenient to perform, adjusts to proband's cognitive ability, and offers a composite outcome. This outcome is sensitive enough to detect age-related cognitive decline in healthy adults, differentiate healthy aging from CNS diseases (MS, NIND, OIND) and differentiate mild cognitive dysfunction observed in CIS and RR-MS from its evolution (P-MS) in chronic CNS disorders such as MS. As digital health technology continues to evolve, our findings support a paradigm shift in cognitive assessment, paving the way for policy changes that prioritize the integration of innovative tools into healthcare systems.

## Data Availability

The original contributions presented in the study are included in the article/Supplementary Material, further inquiries can be directed to the corresponding author.

## Author disclaimer

The contributions of the NIH author(s) were made as part of their official duties as NIH federal employees, are in compliance with agency policy requirements, and are considered Works of the United States Government. However, the findings and conclusions presented in this paper are those of the author(s) and do not necessarily re(ect the views of the NIH or the U.S. Department of Health and Human Services.

## Glossary

BICAMS: brief international cognitive assessment for MS
BPFr: brain parenchymal fraction
BRB-N: brief repeatable battery of neuropsychological tests
BVMT-R: brief visuospatial memory test-retest
CCC: Lin's concordance correlation coefficient
CI: confidence interval
CIS: clinically isolated syndrome
CNS: central nervous system
COMRIS-CTD: combinatorial MRI scale of CNS tissue destruction
CVLT-II: california verbal learning test-II
DelVRI: delayed verbal recall impairment
DelVRLa: delayed verbal recall latency adjusted
DynamicSRI: dynamic spatial recall impairment
DynamicSRLa: dynamic spatial recall latency adjusted
DynamicSRM: dynamic spatial recall mistakes
EDSS: expanded disability status scale
FSS: (Kurtzke) functional system score
FSS7: functional system score 7 (cerebral/mental)
HD: healthy donors
ICC: intraclass correlation coefficient
ImmVRI: immediate verbal recall impairment
ImmVRLa: immediate verbal recall latency adjusted
IPS: information processing speed
iSMPT: individualized sensory-motor processing threshold
iSMPT-S: individualized sensory-motor processing threshold, spatial
iSMPT-V: individualized sensory-motor processing threshold, verbal
JSON: JavaScript object notation
MACFIMS: the minimal assessment of cognitive functions in MS
MRI: magnetic resonance imaging
MS: multiple sclerosis
NeuFun-TS: neurological functions test suite
NeurEx: digital neurological-examination composite score
NeurEx1: cognitive subscore of NeurEx
NIH: national institutes of health
NIAID: national institute of allergy and infectious diseases
NIND: non-inflammatory neurological diseases
OIND: other inflammatory neurological diseases
PAL: paired-associates learning (verbal memory paradigm)
P-MS: progressive multiple sclerosis (SP-MS + PP-MS)
PP-MS: primary-progressive multiple sclerosis
pwMS: people with multiple sclerosis
RLa: recall latencies adjusted
QMENTA: cloud platform for MRI analysis
R²: coefficient of determination
Rho: spearman rank correlation coefficient
RIS: radiologically isolated syndrome
RR-MS: relapsing-remitting multiple sclerosis
SDMT: symbol digit modalities test
SP-MS: secondary-progressive multiple sclerosis
StaticSRI: static spatial recall impairment
StaticSRLa: static spatial recall latency adjusted
StaticSRM: static spatial recall mistakes
VMT: visual matrix task
VPT: visual patters test
VRI: verbl recall impairment
WM: white matter.
