# Supplementary material for: Assessing verbal and spatial memory via smartphone
Source: Front Digit Health. 2026 Mar 10;8:1680389. doi: 10.3389/fdgth.2026.1680389 (PMC13014619; doi:10.3389/fdgth.2026.1680389)
Supplement: Supplementary file 2 [file Presentation1.pdf]

Supplementary Material

1 Supplementary Figures

Supplementary Figure S1

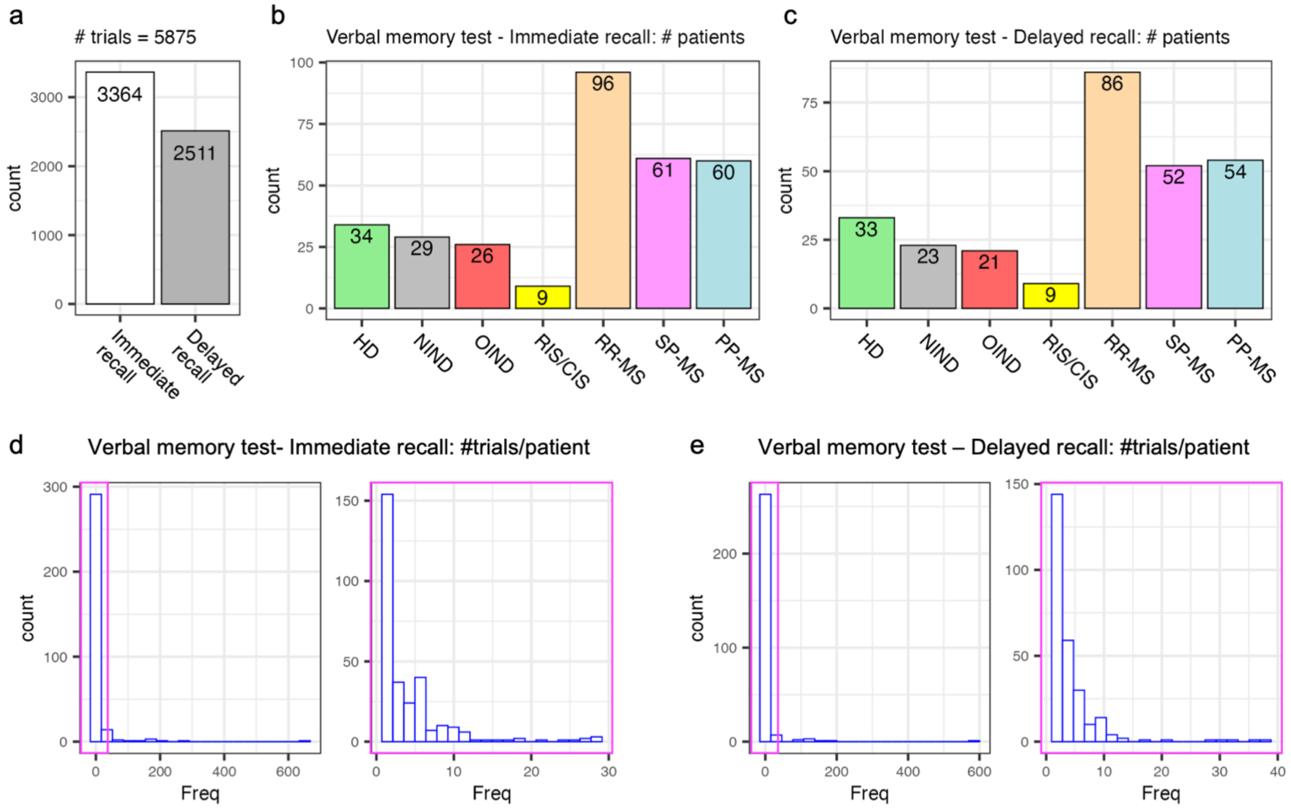

Supplementary Figure S1. Characterization of the verbal memory dataset.
(a) Total trials completed in the immediate recall and delayed recall verbal memory tests.
(b) Number of patients in each diagnostic group (HD = Healthy Donors, NIND = Non-Inflammatory Neurological Diseases, OIND = Other Inflammatory Neurological Diseases, RIS/CIS = Radiologically/Clinically Isolated Syndrome, RR-MS = Relapsing-Remitting MS, SP-MS = Secondary progressive MS, PP-MS = Primary Progressive MS) who completed ≥1 immediate recall trial.
(c) Number of patients in each group who completed ≥1 delayed recall trial.
(d) Distribution of trials per patient in the immediate recall part of the verbal memory test: full range (left) and zoomed-in view of patients with <40 trials (right).
(e) Distribution of trials per patient in the delayed recall part of the verbal memory test: full range; (left) zoomed-in view of patients with <40 trials (right).

Supplementary Figure S2

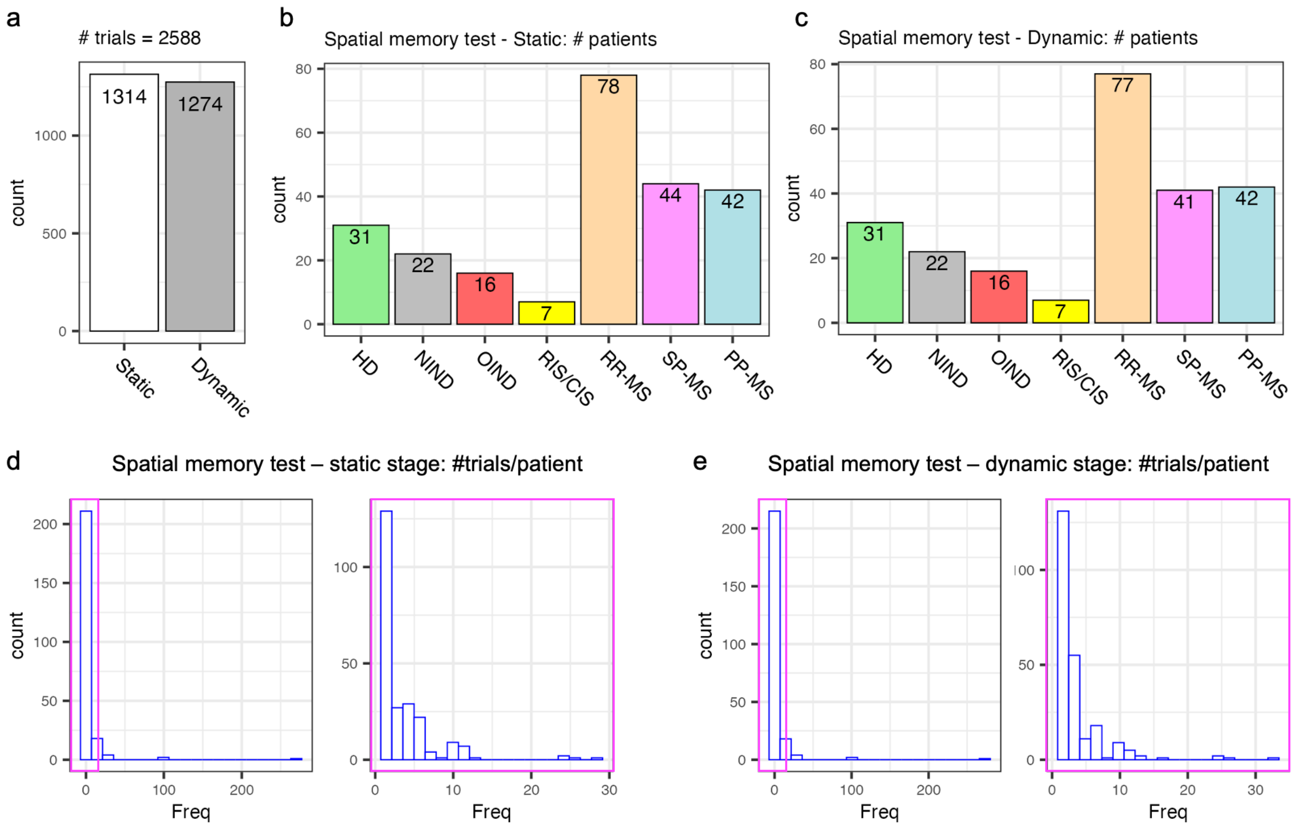

Supplementary Figure S2. Characterization of the spatial memory dataset.
(a) Total trials completed in the static and dynamic spatial memory tests.
(b) Number of patients in each diagnostic group (HD = Healthy Donors, NIND = Non-Inflammatory Neurological Diseases, OIND = Other Inflammatory Neurological Diseases, RIS/CIS = Radiologically/Clinically Isolated Syndrome, RR-MS = Relapsing-Remitting MS, SP-MS = Secondary progressive MS, PP-MS = Primary Progressive MS) who completed ≥1 trial in the static stage of the Spatial memory test.
(c) Number of patients in each group who completed ≥1 trial in the dynamic stage of the Spatial memory test.
(d) Distribution of trials per patient in the static spatial memory test: full range (left) and zoomed-in view of patients with <40 trials (right).
(e) Distribution of trials per patient in the dynamic spatial memory test: full range; (left) zoomed-in view of patients with <40 trials (right).

Supplementary Figure S3

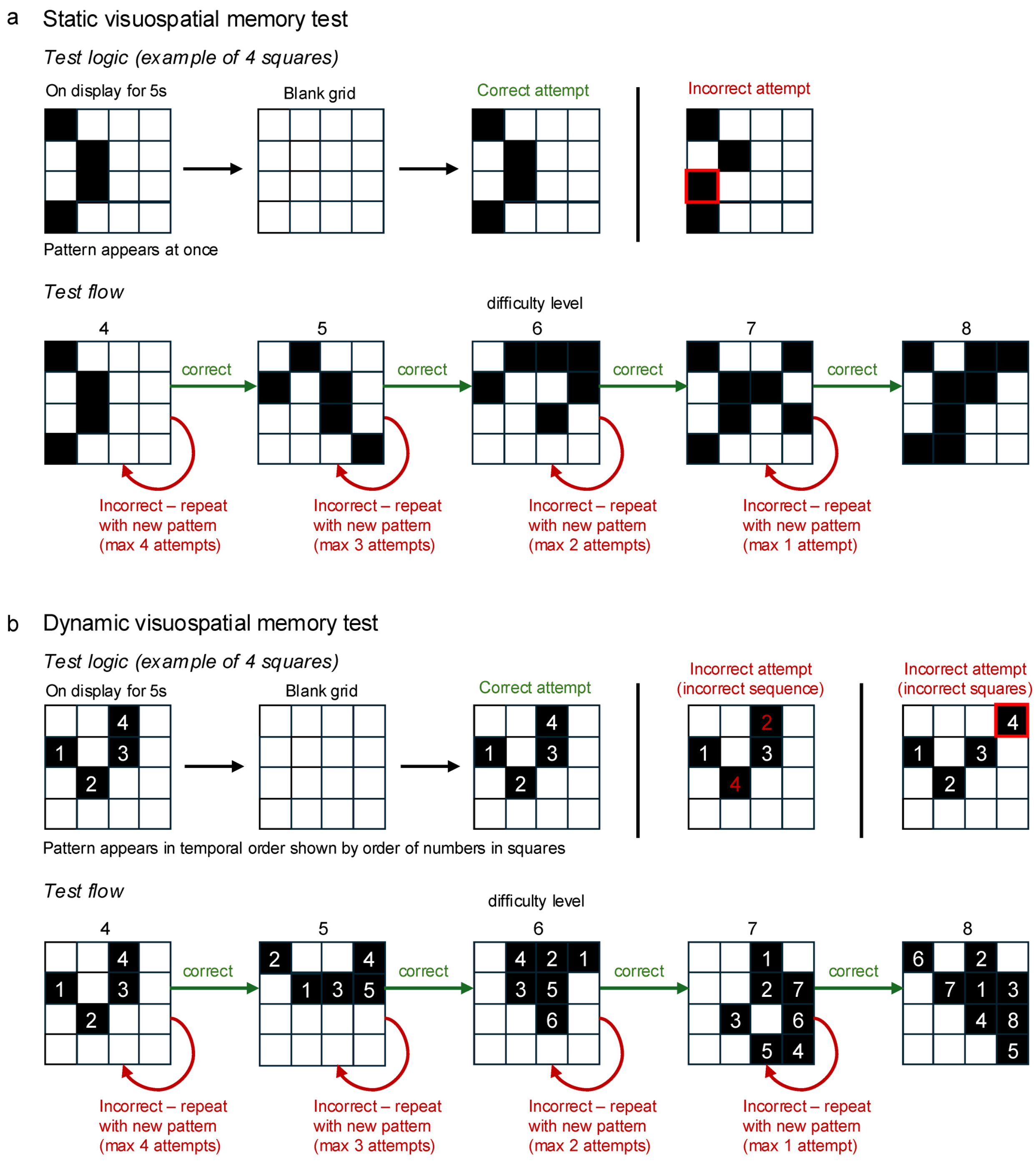

Supplementary Figure S3. Flow diagram of the spatial memory task.
The task consists of a static stage (a) followed by a dynamic stage (b). In the static stage, participants recall spatial patterns of increasing difficulty (4–8 squares), with incorrect responses triggering repetition using a new random configuration and partial-credit scoring applied when a level is not completed. In the dynamic stage, patterns must be reproduced in the correct temporal order; numbers indicate order of square appearance and required recall sequence. Across both stages, maximum of 5 attempts is tested and recorded.

Supplementary Figure S4

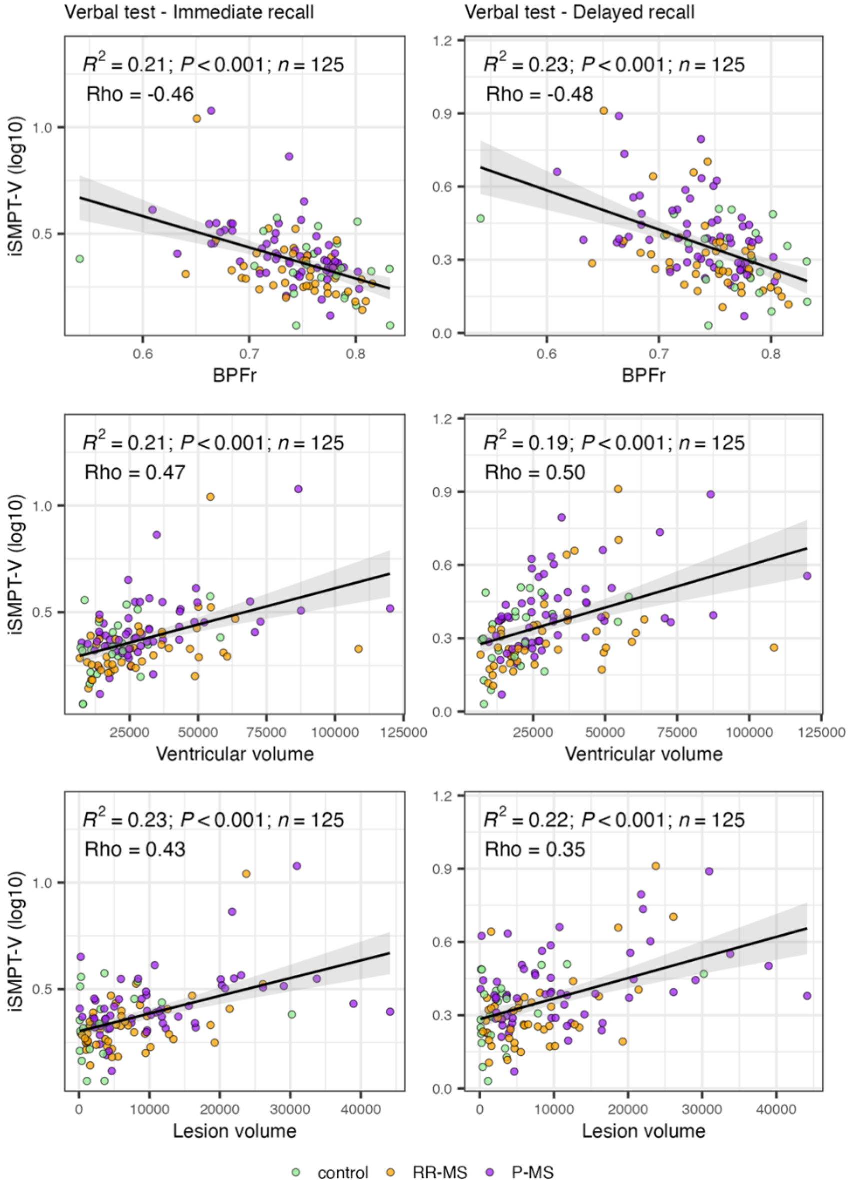

Supplementary Figure S4. Associations between individualized Sensory-Motor Processing Threshold verbal (iSMPT-V) and volumetric MRI measures across verbal memory tests. Verbal memory: first-trial iSMPT-V plotted against Brain Parenchymal Fraction (BPFr), ventricular volume and lesion volume in immediate recall (left) and delayed recall (right).

Points represent controls (all non-MS patients, green), relapsing-remitting MS (RR-MS, orange) and progressive MS (P-MS, purple). Each panel reports the linear fit's coefficient of determination (R^2), its p-value (P) and sample size (n) above the trend line, with the Spearman correlation coefficient (Rho) shown just below.

Confidence intervals for all metrics are in Supplementary Table S8.

Supplementary Figure S5

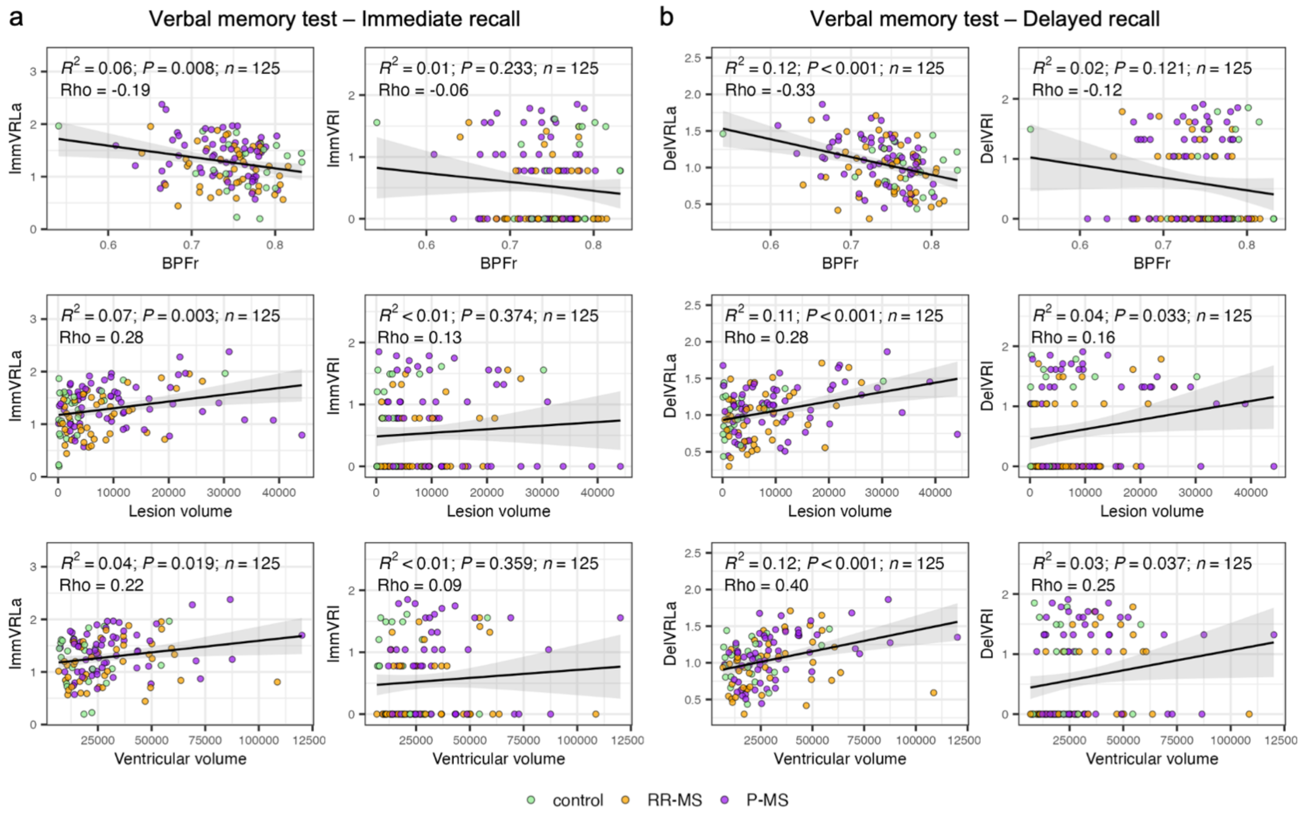

Supplementary Figure S5. Immediate/Delayed Verbal Recall Latency adjusted (ImmVRLa/DelVRLa) and Immediate/Delayed Verbal Recall Impairment (ImmVRI/DelVRI) versus volumetric MRI measures in the Verbal memory test.
(a) Verbal memory Immediate recall stage: first-trial ImmVRLa (left) and ImmVRI (right) plotted against brain parenchymal fraction (BPFr), lesion volume, and ventricular volume. **(b)** Verbal memory Delayed recall stage: first-trial DelVRLa (left) and DelVRI (right) plotted against the same three MRI outcomes.

Points represent controls (all non-MS patients, green), relapsing-remitting MS (RR-MS, orange) and progressive MS (P-MS, purple). Each panel reports the linear fit's coefficient of determination (R^2), its p-value (P) and sample size (n) above the trend line, with the Spearman correlation coefficient (Rho) shown just below.

Confidence intervals for all metrics are in Supplementary Table S9.

Supplementary Figure S6

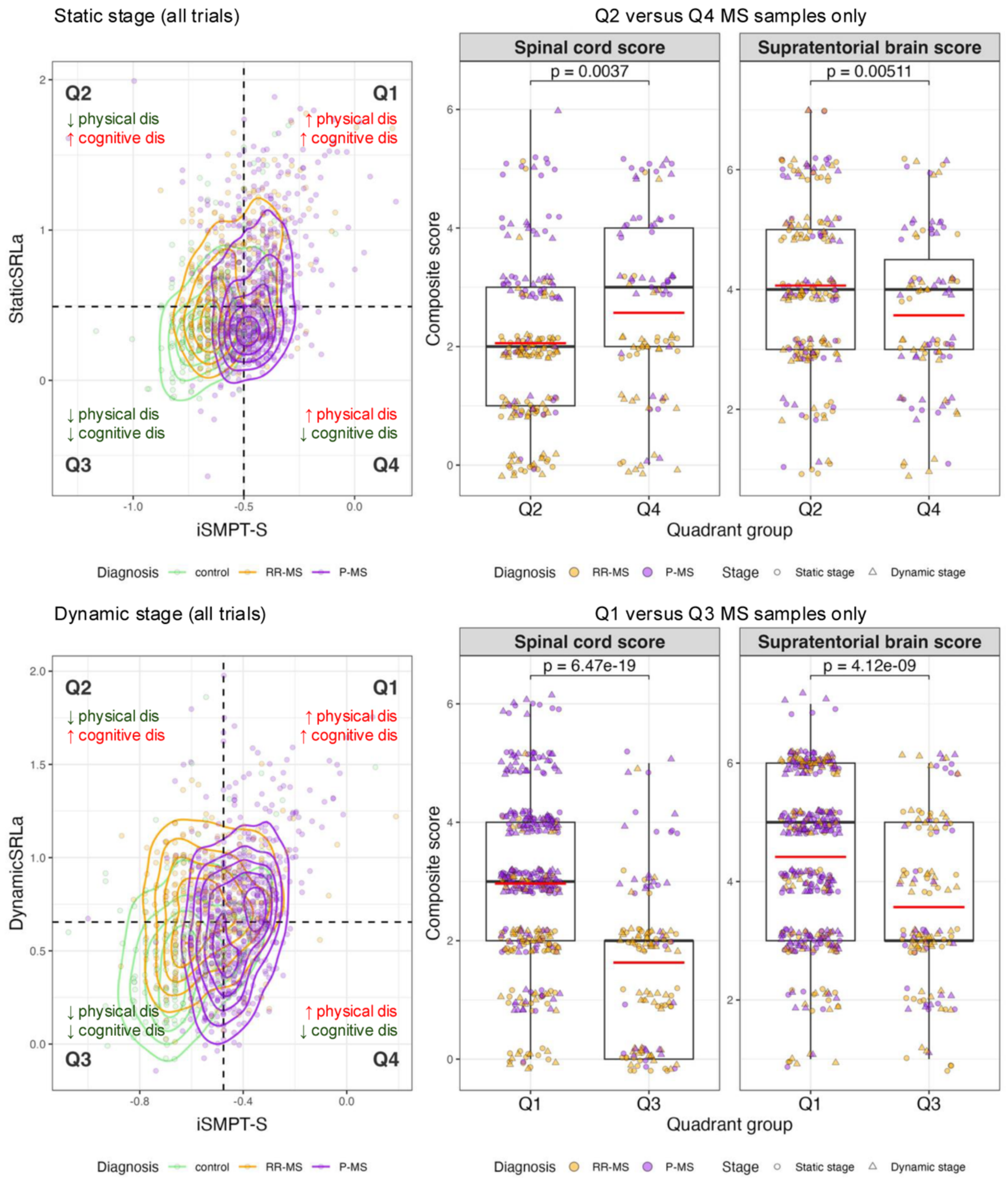

Supplementary Figure S6. Quadrant-based dissociation of sensory-motor and cognitive processing components using iSMPT-S and adjusted recall latency.

Left panels show the joint distribution of individualized Sensory-Motor Processing Threshold derived from the spatial task (iSMPT-S) and adjusted recall latency (StaticSRLa and DynamicSRLa) across all trials. Data from healthy donors and participants with multiple sclerosis are shown, with density contours overlaid by diagnostic group. Dashed vertical and horizontal lines indicate median splits computed across the combined cohort, defining four quadrants: Q1 (high physical and high cognitive disability), Q2 (low physical but high cognitive disability), Q3 (low physical and low

cognitive disability), and Q4 (high physical but relatively preserved cognitive function). Quadrant labels are shown in bold within each region.

Right panels compare imaging-derived composite scores between selected quadrant pairs after restricting the analysis to participants with multiple sclerosis. Boxplots display the distribution of supratentorial brain and spinal cord composite scores within each quadrant group. Boxes represent the interquartile range (IQR), center lines indicate the median, and whiskers extend to 1.5×IQR. Individual observations are overlaid as jittered points, colored by MS subtype and shaped by task stage (static or dynamic). Horizontal red bars indicate the mean composite score within each quadrant and stage.

The spinal cord composite score was calculated as the sum of semi-quantitative COMRIS metrics reflecting lesion load and atrophy in the medulla and upper cervical spinal cord. The supratentorial brain composite score was calculated as the sum of semi-quantitative COMRIS scores for brain T2 lesion load and supratentorial brain atrophy.

Statistical comparisons between quadrant groups were performed using two-sided Wilcoxon rank-sum tests separately for each composite score. Raw p-values are displayed above each boxplot facet; no multiple-comparison correction was applied, as these analyses are intended to illustrate anatomical dissociation patterns rather than to support definitive inferential claims.

Together, these analyses demonstrate that participants classified in Q4 - characterized by elevated iSMPT-S but low adjusted recall latency - exhibit significantly greater spinal cord involvement with relatively preserved supratentorial brain tissue, whereas participants with elevated adjusted recall latency but lower iSMPT-S show the opposite pattern. This supports the interpretation of iSMPT-S as a marker of sensory-motor processing load and adjusted recall latency as a selective indicator of cognitive slowing independent of motor disability.

Supplementary Figure S7

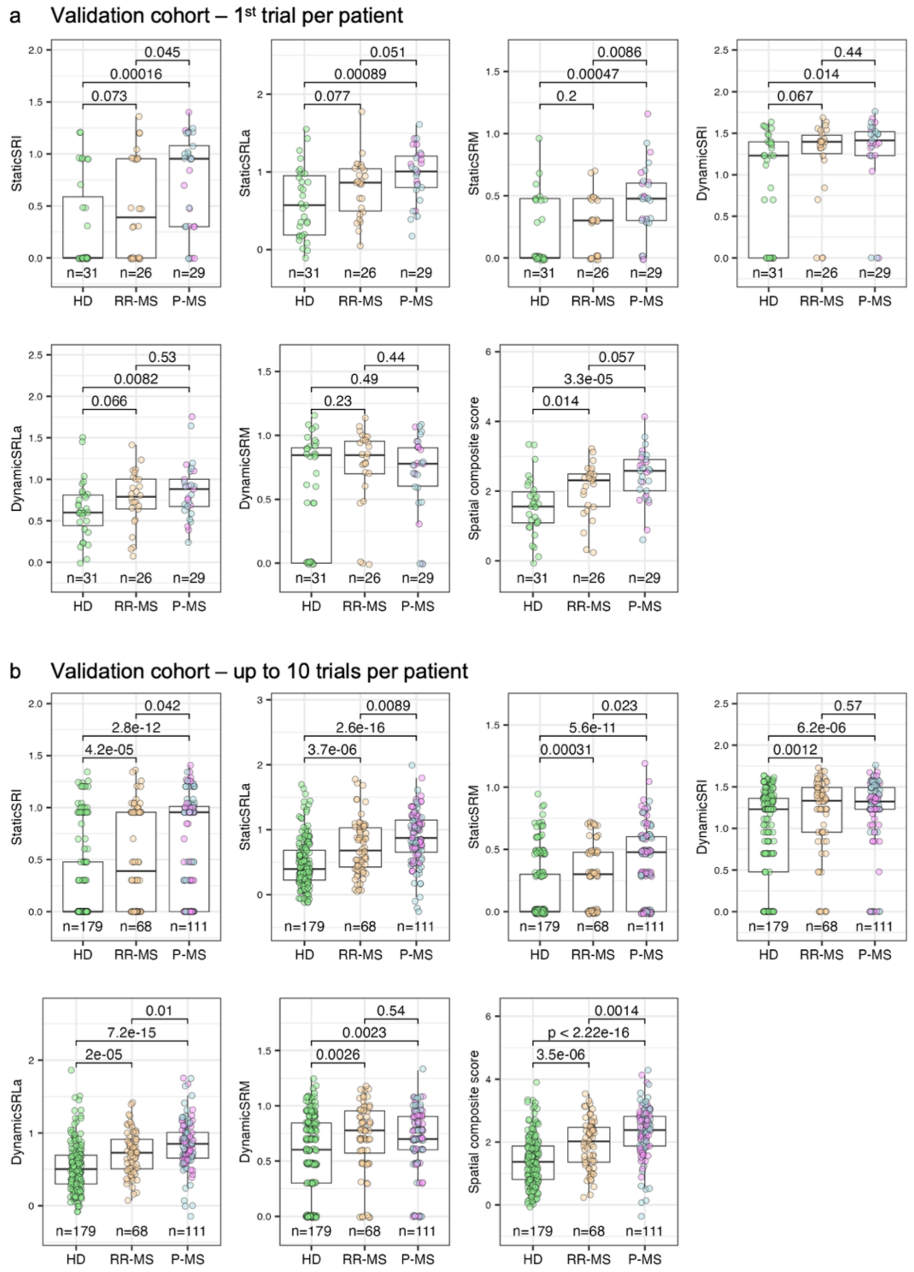

Supplementary Figure S7. Comparison of spatial memory metrics and composite score across diagnostic groups in the validation cohort.

Boxplots of seven measures – Static/Dynamic Spatial Recall Impairment (StaticSRI/DynamicSRI); Static/Dynamic Spatial Recall Latency adjusted (SaticSRLa/DynamicSRLa); Static/Dynamic Spatial RecallMistakes (StaticSRM/DynamicSRM); and the overall spatial composite score—are shown for healthy donors (HD), relapsing-remitting MS (RR-MS) and progressive MS (P-MS). Sample sizes (n) appear below each box. Top panels (a): Validation cohort - first trial per subject. Bottom panels (b): Validation cohort - up to 10 consecutive trials per subject. Wilcoxon rank-sum p-values are annotated above each comparison.

Supplementary Figure S8

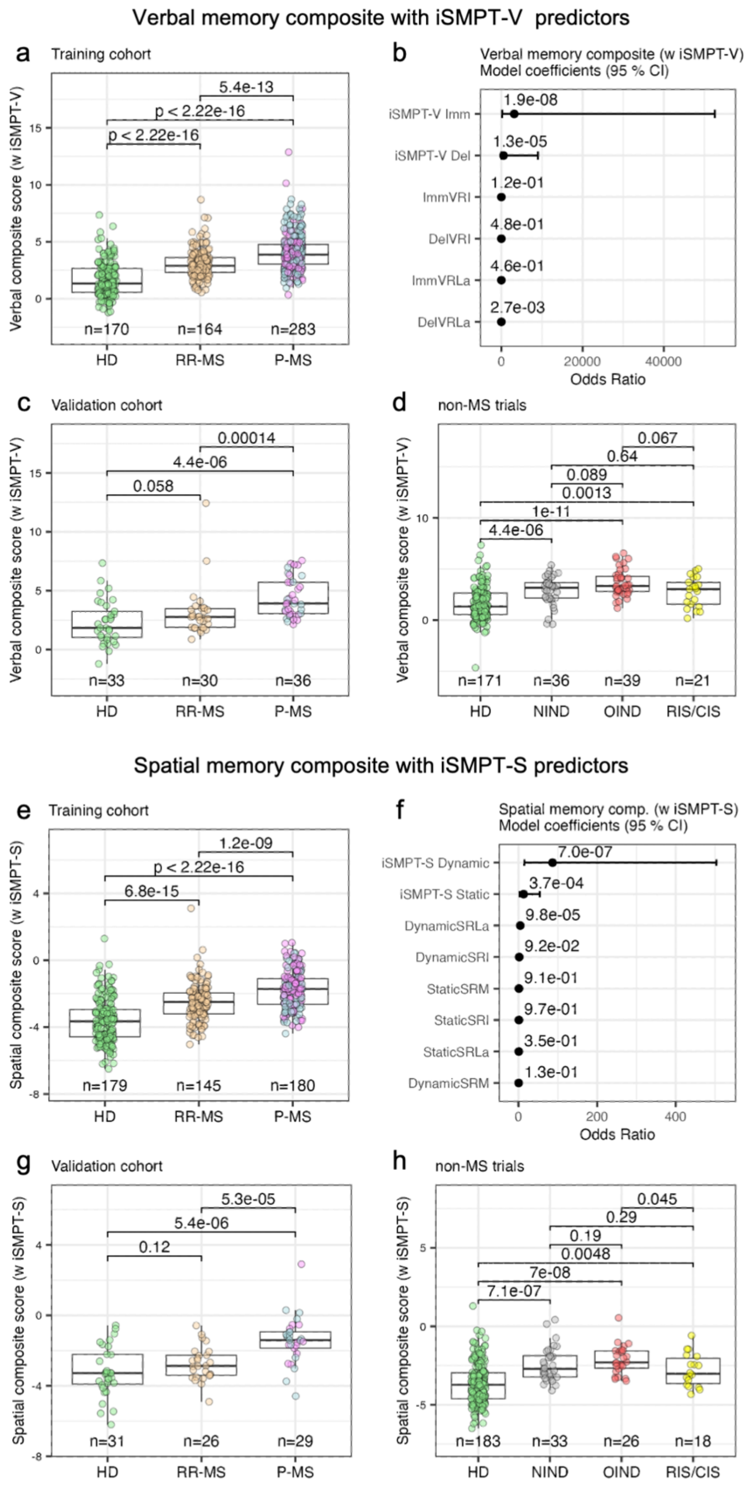

Supplementary Figure S8. Verbal and spatial composite score distributions and model coefficients using individualized Sensory-Motor Processing Threshold Verbal (iSMPT-V) and Spatial (iSMPT-S) predictors.

Verbal composite (panels a–d): (a) Training cohort (up to 10 consecutive trials per subject): boxplots of composite scores for healthy donors (HD), relapsing-remitting MS (RR-MS) and progressive MS

(P-MS); sample sizes (n) are annotated and pair-wise Wilcoxon rank-sum P-values are shown. (b) Ordinal logistic regression: odds ratios (points) and 95 % confidence intervals (bars) for six predictors Immediate/Delayed iSMPT-V (iSMPT-V Imm/ iSMPT-V Del), Immediate/Delayed Verbal Recall Latency adjusted (ImmVRLa/DelVRLa) and Immediate/Delayed Verbal Recall Impairment (ImmVRI/DeVRI). (c) Validation cohort (first trial per subject): composite score distributions by diagnosis with Wilcoxon rank-sum P-values. (d) Non-MS samples: composite scores in HD, non-inflammatory neurological disease (NIND), other inflammatory neurological disease (OIND) and RIS/CIS; Wilcoxon rank-sum p-values are shown.

Spatial composite (panels e–h): (e) Training cohort (up to 10 trials per subject): boxplots for HD, RR-MS and P-MS; sample sizes and Wilcoxon rank-sum P-values are shown. (f) Ordinal logistic regression: odds ratios and 95 % CI for eight predictors Static/Dynamic iSMPT-S; Static/Dynamic Spatial Recall Latency adjusted (StaticSRLa/DynamicSRLa); Static/Dynamic Spatial Recall Impairment (StaticSRI/DynamicSRI); and Static/Dynamic Spatial Recall Mistakes (StaticSRM/DynamicSRM). (g) Validation cohort (first trial/subject): composite score distributions with Wilcoxon rank-sum P-values. (h) Non-MS samples: composite scores in HD, NIND, OIND and RIS/CIS; Wilcoxon rank-sum p-values are shown. Confidence intervals for all metrics are in Supplementary Table S10.

Supplementary Figure S9

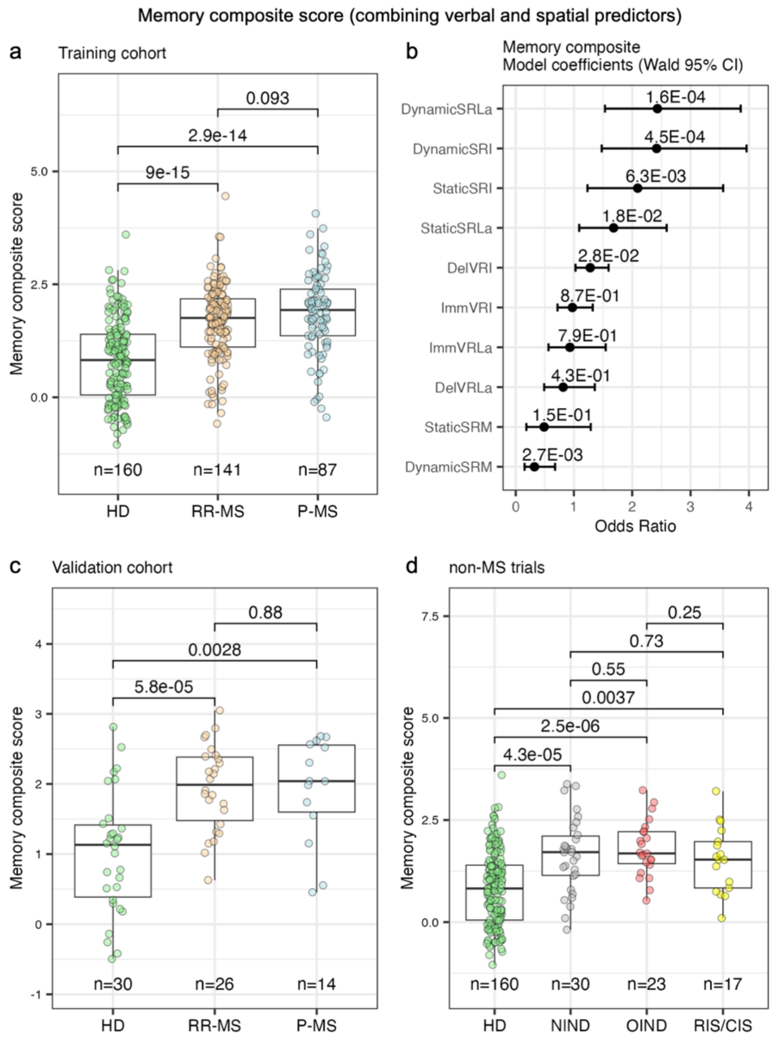

Supplementary Figure S9. Global memory composite score distributions and combined test model coefficients.
(a) Training cohort (up to 10 consecutive trials per subject): boxplots of composite scores for healthy donors (HD), relapsing-remitting MS (RR-MS) and progressive MS (P-MS); sample sizes (n) are shown, and Wilcoxon rank-sum test p-values are annotated. (b) Ordinal logistic regression: odds ratios (points) and 95 % confidence intervals (bars) for ten predictors drawn from both verbal (Immediate/Delayed Verbal Recall Impairment [ImmVRI/DelVRI], Immediate/Delayed Verbal Recall Latency adjusted [ImmVRLa/DelVRLa]) and spatial (Static/Dynamic Spatial Recall Impairment [StaticSRI/DynamicSRI], Static/Dynamic Spatial Recall Latency adjusted [StaticSRLa/DynamicSRLa], Static/Dynamic Spatial Recall Mistakes [StaticSRM/DynamicSRM]) memory tests. (c) Validation cohort (first trial per subject): composite score distributions by diagnosis with Wilcoxon rank-sum P-values. (d) Non-MS cohort: composite scores for HD, non-inflammatory neurological disease (NIND), other inflammatory neurological disease (OIND) and radiologically/clinically isolated syndrome (RIS/CIS); Wilcoxon rank-sum test P-values are shown. Confidence intervals for all metrics are in Supplementary Table S11.

Supplementary Figure S10

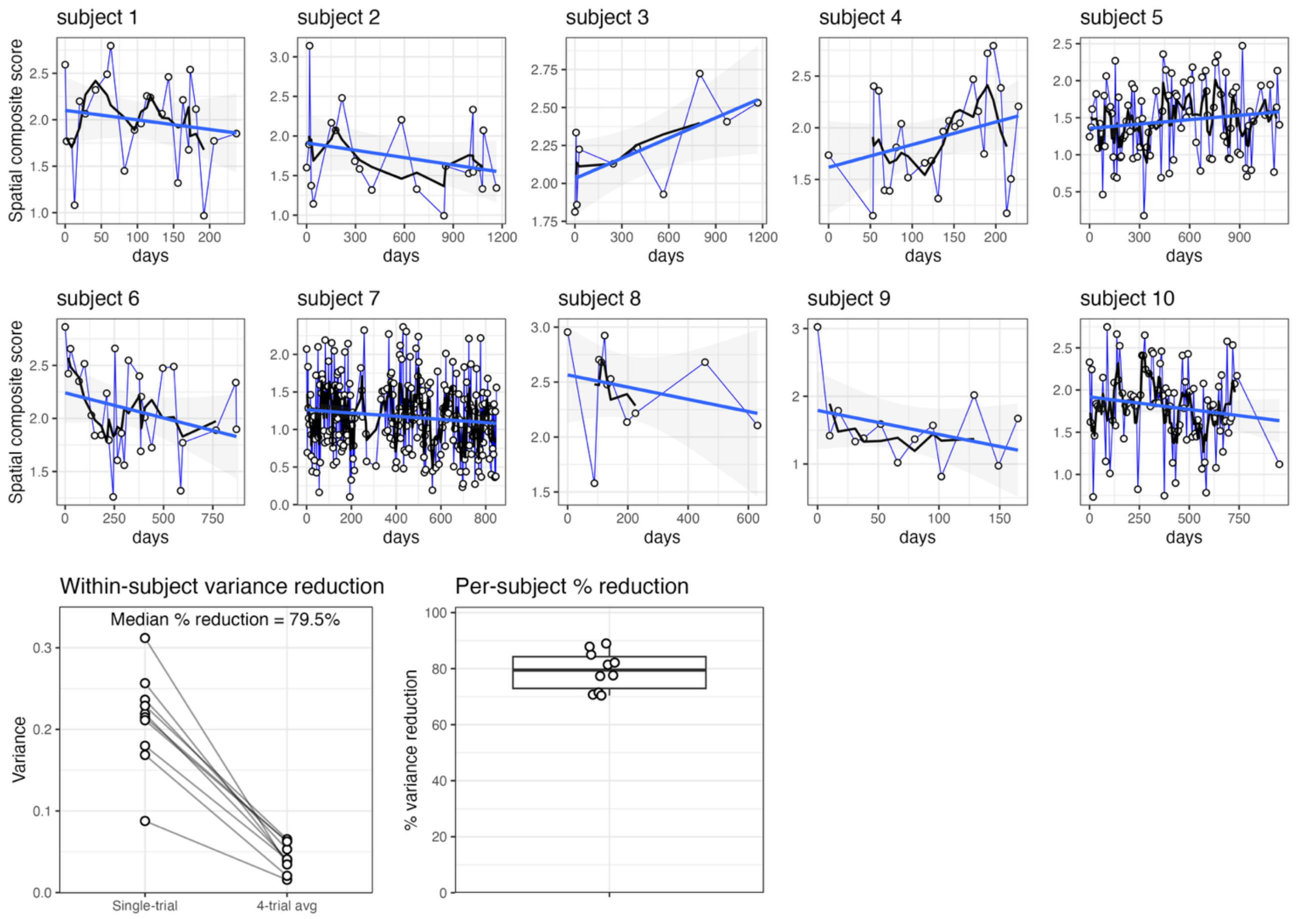

Supplementary Figure S10. Longitudinal spatial composite trajectories and temporal averaging in Cohort 3.

Longitudinal trajectories of the spatial memory composite score in 10 participants with dense repeated testing are shown in the top panels (subjects 1–10). Open circles connected by thin blue lines represent single-trial measurements plotted over days since first assessment. Black lines indicate four-trial rolling averages, illustrating temporal smoothing of short-term variability. Solid blue lines with shaded bands represent linear trends over time with corresponding confidence intervals.

Bottom left panel shows within-subject variance for each participant calculated from single-trial measurements versus variance after four-trial averaging, with lines connecting paired values for the same individual. Bottom right panel summarizes the percent reduction in within-subject variance across participants following temporal averaging. Median variance reduction was 79.5%, demonstrating substantial improvement in signal stability with repeated sampling.

2 Supplementary Tables

Supplementary Table S1: 95% confidence intervals for Spearman's rank correlation coefficient (Rho) and coefficient of determination (R^2), reported in Figure 2.

test	stage	predictor	n	Spearman Rho [95% CI]	R2 [95% CI]
Verbal	Immediate_recall	Age	278	0.446 [0.336, 0.546]	0.151 [0.065, 0.267]
Verbal	Delayed_recall	Age	278	0.456 [0.347, 0.551]	0.159 [0.073, 0.267]
Verbal	Immediate_recall	NeurEx	252	0.487 [0.387, 0.581]	0.228 [0.127, 0.351]
Verbal	Delayed_recall	NeurEx	252	0.552 [0.459, 0.634]	0.297 [0.190, 0.418]
Verbal	Immediate_recall	EDSS	252	0.489 [0.387, 0.580]	0.235 [0.156, 0.327]
Verbal	Delayed_recall	EDSS	252	0.550 [0.458, 0.628]	0.300 [0.218, 0.385]
Verbal	Immediate_recall	COMRIS-CDT	240	0.452 [0.337, 0.556]	0.235 [0.144, 0.333]
Verbal	Delayed_recall	COMRIS-CDT	240	0.434 [0.317, 0.540]	0.234 [0.137, 0.340]
Spatial	Static_visuospatial	Age	236	0.292 [0.174, 0.407]	0.056 [0.013, 0.133]
Spatial	Dynamic_visuospatial	Age	236	0.313 [0.194, 0.427]	0.069 [0.018, 0.154]
Spatial	Static_visuospatial	NeurEx	211	0.480 [0.370, 0.585]	0.157 [0.024, 0.331]
Spatial	Dynamic_visuospatial	NeurEx	211	0.439 [0.324, 0.543]	0.153 [0.057, 0.297]
Spatial	Static_visuospatial	EDSS	211	0.479 [0.365, 0.584]	0.183 [0.066, 0.324]
Spatial	Dynamic_visuospatial	EDSS	211	0.433 [0.316, 0.539]	0.175 [0.089, 0.279]
Spatial	Static_visuospatial	COMRIS-CDT	206	0.309 [0.179, 0.432]	0.079 [0.019, 0.176]
Spatial	Dynamic_visuospatial	COMRIS-CDT	206	0.288 [0.149, 0.417]	0.088 [0.024, 0.184]

Supplementary Table S2: 95% confidence intervals for Spearman's rank correlation coefficient (Rho) and coefficient of determination (R^2), reported in Figure 3.

test	outcome	predictor	n	Spearman Rho [95% CI]	R2 [95% CI]
Verbal	ImmVRLa	NeurEx	252	0.426 [0.309, 0.528]	0.194 [0.117, 0.279]
Verbal	ImmVRLa	NeurEx1 subscore	252	0.419 [0.299, 0.521]	0.181 [0.097, 0.271]
Verbal	ImmVRLa	EDSS	252	0.422 [0.304, 0.528]	0.190 [0.102, 0.289]
Verbal	ImmVRLa	FSS7	252	0.422 [0.313, 0.520]	0.200 [0.114, 0.294]
Verbal	ImmVRLa	COMRIS-CDT	240	0.346 [0.224, 0.457]	0.146 [0.070, 0.239]
Verbal	DelVRLa	NeurEx	252	0.368 [0.255, 0.475]	0.134 [0.062, 0.221]
Verbal	DelVRLa	NeurEx1 subscore	252	0.350 [0.241, 0.463]	0.133 [0.066, 0.214]
Verbal	DelVRLa	EDSS	252	0.373 [0.258, 0.480]	0.161 [0.085, 0.251]
Verbal	DelVRLa	FSS7	252	0.357 [0.246, 0.466]	0.145 [0.075, 0.236]
Verbal	DelVRLa	COMRIS-CDT	240	0.384 [0.268, 0.496]	0.174 [0.097, 0.271]
Verbal	ImmVRI	NeurEx	252	0.350 [0.235, 0.455]	0.130 [0.067, 0.209]
Verbal	ImmVRI	NeurEx1 subscore	252	0.377 [0.272, 0.480]	0.128 [0.069, 0.204]
Verbal	ImmVRI	EDSS	252	0.352 [0.240, 0.456]	0.131 [0.065, 0.212]
Verbal	ImmVRI	FSS7	252	0.371 [0.265, 0.477]	0.145 [0.077, 0.233]
Verbal	ImmVRI	COMRIS-CDT	240	0.226 [0.097, 0.345]	0.057 [0.012, 0.127]
Verbal	DelVRI	NeurEx	252	0.309 [0.197, 0.416]	0.070 [0.021, 0.142]
Verbal	DelVRI	NeurEx1 subscore	252	0.347 [0.240, 0.457]	0.069 [0.017, 0.158]
Verbal	DelVRI	EDSS	252	0.312 [0.200, 0.414]	0.097 [0.041, 0.171]
Verbal	DelVRI	FSS7	252	0.344 [0.235, 0.455]	0.120 [0.055, 0.210]
Verbal	DelVRI	COMRIS-CDT	240	0.276 [0.152, 0.393]	0.068 [0.020, 0.144]

Supplementary Table S3: 95% confidence intervals for Spearman's rank correlation coefficient (Rho) and coefficient of determination (R^2), reported in Figure 4.

test	outcome	predictor	n	Spearman Rho [95% CI]	R2 [95% CI]
Spatial	StaticSRI	NeurEx	211	0.341 [0.209, 0.459]	0.059 [0.013, 0.138]
Spatial	StaticSRI	NeurEx1 subscore	211	0.348 [0.226, 0.463]	0.073 [0.031, 0.158]
Spatial	StaticSRI	EDSS	211	0.360 [0.228, 0.484]	0.103 [0.035, 0.199]
Spatial	StaticSRI	FSS7	211	0.320 [0.199, 0.435]	0.100 [0.038, 0.187]
Spatial	StaticSRI	COMRIS-CDT	206	0.287 [0.148, 0.417]	0.058 [0.010, 0.140]
Spatial	DynamicSRI	NeurEx	211	0.261 [0.136, 0.384]	0.030 [0.002, 0.090]
Spatial	DynamicSRI	NeurEx1 subscore	211	0.272 [0.137, 0.399]	0.046 [0.012, 0.096]
Spatial	DynamicSRI	EDSS	211	0.288 [0.159, 0.411]	0.081 [0.027, 0.160]
Spatial	DynamicSRI	FSS7	211	0.259 [0.124, 0.387]	0.058 [0.012, 0.136]
Spatial	DynamicSRI	COMRIS-CDT	206	0.202 [0.060, 0.329]	0.023 [0.000, 0.086]
Spatial	StaticSRLa	NeurEx	211	0.332 [0.193, 0.456]	0.134 [0.047, 0.252]
Spatial	StaticSRLa	NeurEx1 subscore	211	0.394 [0.276, 0.514]	0.151 [0.083, 0.232]
Spatial	StaticSRLa	EDSS	211	0.328 [0.194, 0.450]	0.129 [0.050, 0.231]
Spatial	StaticSRLa	FSS7	211	0.360 [0.244, 0.479]	0.142 [0.068, 0.241]
Spatial	StaticSRLa	COMRIS-CDT	206	0.380 [0.239, 0.499]	0.156 [0.065, 0.264]
Spatial	DynamicSRLa	NeurEx	211	0.301 [0.165, 0.422]	0.162 [0.060, 0.293]
Spatial	DynamicSRLa	NeurEx1 subscore	211	0.280 [0.147, 0.411]	0.060 [0.015, 0.173]
Spatial	DynamicSRLa	EDSS	211	0.339 [0.210, 0.460]	0.141 [0.060, 0.245]
Spatial	DynamicSRLa	FSS7	211	0.246 [0.109, 0.377]	0.071 [0.019, 0.153]
Spatial	DynamicSRLa	COMRIS-CDT	206	0.332 [0.203, 0.452]	0.134 [0.053, 0.232]
Spatial	StaticSRM	NeurEx	211	0.302 [0.177, 0.423]	0.062 [0.015, 0.141]
Spatial	StaticSRM	NeurEx1 subscore	211	0.328 [0.199, 0.448]	0.057 [0.016, 0.165]
Spatial	StaticSRM	EDSS	211	0.319 [0.188, 0.440]	0.102 [0.039, 0.192]
Spatial	StaticSRM	FSS7	211	0.297 [0.166, 0.420]	0.086 [0.029, 0.170]
Spatial	StaticSRM	COMRIS-CDT	206	0.285 [0.153, 0.410]	0.078 [0.023, 0.162]
Spatial	DynamicSRM	NeurEx	211	0.168 [0.032, 0.298]	0.020 [0.000, 0.082]
Spatial	DynamicSRM	NeurEx1 subscore	211	0.189 [0.045, 0.328]	0.037 [0.004, 0.090]
Spatial	DynamicSRM	EDSS	211	0.189 [0.055, 0.326]	0.043 [0.006, 0.111]
Spatial	DynamicSRM	FSS7	211	0.165 [0.022, 0.307]	0.030 [0.001, 0.100]
Spatial	DynamicSRM	COMRIS-CDT	206	0.183 [0.044, 0.312]	0.025 [0.000, 0.084]

Supplementary Table S4: 95% confidence intervals for odds ratios reported in Figure 5.

Variable	Odds Ratio	Wald-type 95% CI	p-value (Wald)
ImmVRI	0.579	[0.383, 0.874]	9.25E-03
ImmVRLa	2.291	[1.178, 4.455]	1.46E-02
DelVRI	1.168	[0.872, 1.565]	2.98E-01
DelVRLa	1.408	[0.734, 2.702]	3.03E-01

Supplementary Table S5: 95% confidence intervals for odds ratios reported in Figure 6.

Variable	Odds Ratio	Wald-type 95% CI	p-value (Wald)
StaticSRI	1.72	[0.765, 3.869]	1.90E-01
StaticSRLa	1.606	[0.839, 3.076]	1.53E-01
StaticSRM	0.617	[0.144, 2.646]	5.15E-01
DynamicSRI	2.899	[1.429, 5.882]	3.18E-03
DynamicSRLa	5.268	[2.654, 10.459]	2.04E-06
DynamicSRM	0.25	[0.082, 0.761]	1.47E-02

Supplementary Table S6: 95% confidence intervals for Spearman's rank correlation coefficient (Rho), coefficient of determination (R²), and odds ratios reported in Figure 7.

model	term	Odds Ratio	Wald-type 95% CI	p-value (Wald)
Full	StaticSRI	1.866	[-4.073, 7.805]	5.37E-01
Full	StaticSRLa	-3.14	[-8.300, 2.020]	2.32E-01
Full	StaticSRM	-10.5	[-21.020, 0.020]	5.04E-02
Full	iSMPT-S Static	-16.294	[-26.682, -5.906]	2.22E-03
Full	DynamicSRI	-7.283	[-13.097, -1.470]	1.43E-02
Full	DynamicSRLa	-10.669	[-15.916, -5.421]	8.09E-05
Full	DynamicSRM	2.704	[-6.145, 11.553]	5.48E-01
Full	iSMPT-S Dynamic	-13.737	[-26.178, -1.296]	3.06E-02
Reduced	StaticSRM	-9.721	[-14.549, -4.893]	9.42E-05
Reduced	iSMPT-S Static	-16.291	[-26.625, -5.958]	2.11E-03
Reduced	DynamicSRI	-5.864	[-9.734, -1.993]	3.12E-03
Reduced	DynamicSRLa	-11.544	[-15.769, -7.320]	1.60E-07
Reduced	iSMPT-S Dynamic	-15.418	[-27.348, -3.489]	1.15E-02

model	R2 [95% CI]	Adjusted R2 [95% CI]
Full	0.458 [0.380, 0.556]	0.442 [0.361, 0.545]
Reduced	0.453 [0.368, 0.546]	0.443 [0.357, 0.542]

cohort	n	Spearman Rho [95% CI]	R2 [95% CI]
Training	280	0.642 [0.558, 0.713]	0.453 [0.360, 0.540]
Validation	63	0.739 [0.596, 0.833]	0.534 [0.378, 0.670]

Supplementary Table S7: 95% confidence intervals for Spearman's rank correlation coefficient (Rho), coefficient of determination (R^2), intraclass correlation coefficient (ICC), reported in Figure 8.

variable	n patients	n observations	ICC [95% CI]
Spatial composite score	86	148	0.713 [0.606, 0.789]
Spatial composite score (w iSMPT-S)	86	148	0.813 [0.689, 0.884]
Spatial memory-predicted SDMT	86	148	0.820 [0.740, 0.873]

variable	n	Spearman Rho [95% CI]	R2 [95% CI]
Spatial composite score	32	0.353 [-0.042, 0.692]	0.135 [0.003, 0.405]
Spatial composite score (w iSMPT-S)	32	0.667 [0.398, 0.840]	0.445 [0.236, 0.643]
Spatial memory-predicted SDMT	32	-0.552 [-0.788, -0.231]	0.373 [0.138, 0.642]

Supplementary Table S8: 95% confidence intervals for Spearman's rank correlation coefficient (Rho), and coefficient of determination (R^2), reported in Supplementary Figure S4.

test	stage	predictor	n	Spearman Rho [95% CI]	R2 [95% CI]
Verbal	Immediate_recall	BPFr	111	-0.429 [-0.575, -0.262]	0.190 [0.078, 0.341]
Verbal	Delayed_recall	BPFr	111	-0.451 [-0.581, -0.292]	0.203 [0.090, 0.326]
Verbal	Immediate_recall	Lesion volume	111	0.419 [0.229, 0.572]	0.194 [0.089, 0.333]
Verbal	Delayed_recall	Lesion volume	111	0.351 [0.149, 0.524]	0.196 [0.082, 0.339]
Verbal	Immediate_recall	Ventricular volume	111	0.431 [0.254, 0.585]	0.142 [0.059, 0.268]
Verbal	Delayed_recall	Ventricular volume	111	0.464 [0.303, 0.598]	0.142 [0.048, 0.281]
Spatial	Static_visuospatial	BPFr	33	-0.155 [-0.484, 0.205]	0.041 [0.000, 0.230]
Spatial	Dynamic_visuospatial	BPFr	33	-0.161 [-0.500, 0.193]	0.011 [0.000, 0.208]
Spatial	Static_visuospatial	Lesion volume	33	0.178 [-0.195, 0.505]	0.075 [0.000, 0.355]
Spatial	Dynamic_visuospatial	Lesion volume	33	0.342 [-0.034, 0.634]	0.061 [0.000, 0.424]
Spatial	Static_visuospatial	Ventricular volume	33	0.335 [-0.013, 0.620]	0.106 [0.002, 0.356]
Spatial	Dynamic_visuospatial	Ventricular volume	33	0.200 [-0.206, 0.581]	0.018 [0.000, 0.247]

Supplementary Table S9: 95% confidence intervals for Spearman's rank correlation coefficient (Rho) and coefficient of determination (R^2), reported in Supplementary Figure S5.

outcome	predictor	n	Spearman Rho [95% CI]	R2 [95% CI]
ImmVRLa	BPFr	125	-0.193 [-0.358, -0.009]	0.056 [0.002, 0.152]
ImmVRLa	Lesion volume	125	0.283 [0.100, 0.446]	0.069 [0.004, 0.218]
ImmVRLa	Ventricular volume	125	0.223 [0.041, 0.384]	0.044 [0.001, 0.159]
DelVRLa	BPFr	125	-0.333 [-0.486, -0.162]	0.119 [0.032, 0.239]
DelVRLa	Lesion volume	125	0.275 [0.083, 0.452]	0.113 [0.021, 0.263]
DelVRLa	Ventricular volume	125	0.399 [0.233, 0.542]	0.122 [0.024, 0.271]
ImmVRI	BPFr	125	-0.058 [-0.244, 0.129]	0.012 [0.000, 0.085]
ImmVRI	Lesion volume	125	0.127 [-0.059, 0.300]	0.006 [0.000, 0.072]
ImmVRI	Ventricular volume	125	0.090 [-0.093, 0.264]	0.007 [0.000, 0.073]
DelVRI	BPFr	125	-0.121 [-0.289, 0.063]	0.019 [0.000, 0.094]
DelVRI	Lesion volume	125	0.162 [-0.013, 0.323]	0.036 [0.001, 0.128]
DelVRI	Ventricular volume	125	0.250 [0.083, 0.400]	0.035 [0.001, 0.119]

Supplementary Table S10: 95% confidence intervals for odds ratios reported in Supplementary Figure S8.

Model	Variable	Odds Ratio	Wald-type 95% CI	p-value (Wald)
Verbal (w iSMPT-V)	ImmVRI	1.438	[0.909, 2.274]	1.20E-01
Verbal (w iSMPT-V)	ImmVRLa	0.753	[0.352, 1.610]	4.60E-01
Verbal (w iSMPT-V)	iSMPT-V Imm	3164.172	[190.469, 52564.951]	1.90E-08
Verbal (w iSMPT-V)	DelVRI	1.119	[0.820, 1.529]	4.80E-01
Verbal (w iSMPT-V)	DelVRLa	0.318	[0.151, 0.671]	2.70E-03
Verbal (w iSMPT-V)	iSMPT-V Del	535.577	[31.917, 8987.014]	1.30E-05
Spatial (w iSMPT-S)	StaticSRI	1.016	[0.451, 2.290]	9.70E-01
Spatial (w iSMPT-S)	StaticSRLa	0.72	[0.364, 1.424]	3.50E-01
Spatial (w iSMPT-S)	StaticSRM	1.094	[0.229, 5.237]	9.10E-01
Spatial (w iSMPT-S)	iSMPT-S Static	13.102	[3.182, 53.943]	3.70E-04
Spatial (w iSMPT-S)	DynamicSRI	1.822	[0.907, 3.663]	9.20E-02
Spatial (w iSMPT-S)	DynamicSRLa	4.445	[2.099, 9.413]	9.80E-05
Spatial (w iSMPT-S)	DynamicSRM	0.417	[0.135, 1.286]	1.30E-01
Spatial (w iSMPT-S)	iSMPT-S Dynamic	86.459	[14.851, 503.337]	7.00E-07

Supplementary Table S11: 95% confidence intervals for odds ratios reported in Supplementary Figure S9.

Model	Variable	Odds Ratio	Wald-type 95% CI	p-value (Wald)
Memory composite	StaticSRI	2.094	[1.233, 3.558]	6.30E-03
Memory composite	StaticSRLa	1.682	[1.092, 2.590]	1.80E-02
Memory composite	StaticSRM	0.488	[0.185, 1.289]	1.50E-01
Memory composite	DynamicSRI	2.418	[1.477, 3.959]	4.50E-04
Memory composite	DynamicSRLa	2.432	[1.533, 3.858]	1.60E-04
Memory composite	DynamicSRM	0.323	[0.154, 0.676]	2.70E-03
Memory composite	ImmVRI	0.975	[0.718, 1.323]	8.70E-01
Memory composite	ImmVRLa	0.933	[0.563, 1.545]	7.90E-01
Memory composite	DelVRI	1.28	[1.027, 1.594]	2.80E-02
Memory composite	DelVRLa	0.816	[0.490, 1.358]	4.30E-01

Supplementary Table S12: Ordinal logistic regression coefficients for the spatial-memory composite models

Spatial memory composite with iSMPT-S predictors

Predictor	Log-odds (β)	Std. Error	Odds Ratio (OR)	OR 95% CI (low)	OR 95% CI (high)	P (Wald)
iSMPT-S Dynamic	4.460	0.899	86.459	14.851	503.321	6.98E-07
iSMPT-S Static	2.573	0.722	13.102	3.183	53.941	3.66E-04
DynamicSRLa	1.492	0.383	4.445	2.099	9.413	9.78E-05
DynamicSRI	0.600	0.356	1.822	0.907	3.663	9.21E-02
StaticSRM	0.090	0.799	1.094	0.229	5.236	9.10E-01
StaticSRI	0.016	0.415	1.016	0.451	2.290	9.70E-01
StaticSRLa	-0.328	0.348	0.720	0.364	1.424	3.45E-01
DynamicSRM	-0.876	0.575	0.417	0.135	1.286	1.28E-01
RRMS|PMS	-1.818	0.557	0.162	0.054	0.484	1.10E-03
HD|RRMS	-3.528	0.615	0.029	0.009	0.098	9.48E-09

Spatial memory composite without iSMPT-S predictors

Predictor	Log-odds (β)	Std. Error	Odds Ratio (OR)	OR 95% CI (low)	OR 95% CI (high)	P (Wald)
RRMS|PMS	2.578	0.273	13.173	7.713	22.500	0.00E+00
DynamicSRLa	1.662	0.350	5.268	2.654	10.459	2.04E-06
HD|RRMS	1.212	0.254	3.359	2.042	5.524	1.81E-06
DynamicSRI	1.065	0.361	2.899	1.429	5.882	3.18E-03
StaticSRI	0.543	0.414	1.720	0.765	3.869	1.90E-01
StaticSRLa	0.474	0.332	1.606	0.839	3.076	1.53E-01
StaticSRM	-0.484	0.743	0.617	0.144	2.646	5.15E-01
DynamicSRM	-1.388	0.569	0.250	0.082	0.761	1.47E-02

Supplementary Table S13: Confusion matrices for logistic regression models in the independent validation cohort

Spatial memory composite

Accuracy: 51.2%		Predicted diagnosis		
		**HD**	**RRMS**	**PMS**
Actual diagnosis	**HD**	22	0	9
	**RRMS**	8	0	18
	**PMS**	7	0	22

Spatial memory composite with iSMPT-S predictors

Accuracy: 61.6%		Predicted diagnosis		
		**HD**	**RRMS**	**PMS**
Actual diagnosis	**HD**	20	4	7
	**RRMS**	9	11	6
	**PMS**	2	5	22

Verbal memory composite

Accuracy: 39.4%		Predicted diagnosis		
		**HD**	**RRMS**	**PMS**
Actual diagnosis	**HD**	3	0	30
	**RRMS**	2	0	28
	**PMS**	0	0	36

Verbal memory composite with iSMPT-V predictors

Accuracy: 55.6%		Predicted diagnosis		
		**HD**	**RRMS**	**PMS**
Actual diagnosis	**HD**	17	7	9
	**RRMS**	8	11	11
	**PMS**	0	9	27
